# Antibacterial Activities of Azole Complexes Combined with Silver Nanoparticles

**DOI:** 10.3390/molecules23020361

**Published:** 2018-02-08

**Authors:** Nestor J. Bello-Vieda, Homero F. Pastrana, Manuel F. Garavito, Alba G. Ávila, Adriana M. Celis, Alvaro Muñoz-Castro, Silvia Restrepo, John J. Hurtado

**Affiliations:** 1 Department of Chemistry, Universidad de los Andes, Carrera 1 No. 18A-12, Bogotá 111711, Colombia; nj.bello1211@uniandes.edu.co; 2 Eléctrica y Electrónica, Centro de Microelectrónica, Universidad de Los Andes, Carrera 1 No. 18A-12, Bogotá 111711, Colombia; hf.pastrana122@uniandes.edu.co (H.F.P.); a-avila@uniandes.edu.co (A.G.Á.); 3 Laboratorio de Micología y Fitopatología, Departamento de Ciencias Biológicas, Universidad de Los Andes, Carrera 1 No. 18A-12, Bogotá 111711, Colombia; manuel.garavitodiago@yale.edu (M.F.G.); acelis@uniandes.edu.co (A.M.C.); srestrep@uniandes.edu.co (S.R.); 4 Grupo de Química Inorgánica y Materiales Moleculares, Universidad Autonoma de Chile, El Llano Subercaseaux 2801, Santiago, Chile; alvaro.munoz@uautonoma.cl

**Keywords:** azole ligands, copper and cobalt complexes, silver nanoparticles, antibacterial resistance, antibacterial activity, cytotoxicity

## Abstract

Growing antimicrobial resistance is considered a potential threat for human health security by health organizations, such as the WHO, CDC and FDA, pointing to MRSA as an example. New antibacterial drugs and complex derivatives are needed to combat the development of bacterial resistance. Six new copper and cobalt complexes of azole derivatives were synthesized and isolated as air-stable solids and characterized by melting point analyses, elemental analyses, thermogravimetric analyses (TGA), and infrared and ultraviolet/visible spectroscopy. The analyses and spectral data showed that the complexes had 1:1 (M:L) stoichiometries and tetrahedral geometries, the latter being supported by DFT calculations. The antibacterial activities of the metal complexes by themselves and combined with silver nanoparticles (AgNPs; 2 μg mL^−1^) were assessed in vitro by broth microdilution assays against eight bacterial strains of clinical relevance. The results showed that the complexes alone exhibited moderate antibacterial activities. However, when the metal complexes were combined with AgNPs, their antibacterial activities increased (up to 10-fold in the case of complex **5**), while human cell viabilities were maintained. The minimum inhibitory concentration (MIC_50_) values were in the range of 25–500 μg mL^−1^. This study thus presents novel approaches for the design of materials for fighting bacterial resistance. The use of azole complexes combined with AgNPs provides a new alternative against bacterial infections, especially when current treatments are associated with the rapid development of antibiotic resistance.

## 1. Introduction

The bioactivity of some metal nanoparticles and their uses in diagnostic sensors are research areas of growing interest [1]. Nanosized metal particles show notable physical, chemical and biological properties compared with bulk solids [2]. Silver nanoparticles (AgNPs) have attracted the interest of the scientific community because silver has been used as an antiseptic and antimicrobial against different species of bacteria [1,2]. The growing resistance of pathogenic bacterial and fungal strains to traditional treatments is another key factor driving research with this type of material. In recent years, the effectiveness of some nanoconjugates has been demonstrated against some pathogenic microbes, showing great potential for nanomedicine. The combination of antibiotics, antifungal agents such as azoles, metal complexes and nanoparticles could increase the efficiency of drugs, even against drug-resistant pathogens [3,4].

AgNPs have been used in recent years for the production of a new class of antimicrobials to combat a widespread range of pathogens. Even though there have been several studies showing the high antibacterial effect of AgNPs, their mechanism of action is not fully understood [5]. The broad activity spectrum of AgNPs against morphologically and metabolically diverse microorganisms could be related to a complex and multilayered mechanism of action. Several mechanisms have been proposed and include the alteration of cell walls and cytoplasm. Among these mechanisms are the membrane permeability increase and respiration depletion, morphological changes of the cytoskeleton, the separation of the cytoplasmic membrane from the cell wall, and plasmolysis and inhibition of bacterial DNA replication [2]. Nevertheless, the high bactericidal activity is related to doses over 40 μg mL^−1^, but these doses have been reported as cytotoxic in mammalian cells by Zhang et al. [6] and toxic on aquatic organisms such as algae by Sass et al. [7]. Better antibacterial activity with low AgNP loadings can be achieved by the addition of compounds with low resistance such as metal azole complexes, limiting the potential toxicity of nanoparticles. These compounds are produced using a metallic salt and an appropriate ligand with chelating properties. The use of azole derivatives as a ligand is an interesting approach because azoles have antifungal and antibacterial activities [8]. These activities are related to their ability to bind readily to enzymes and receptors in biological systems. When the azole ligand is coordinated to Co^II^ or Cu^II^, these complexes have higher antimicrobial activity than the free ligand, and in some cases, they exceed that of standard test substances [9,10,11,12].

The combined activity of metal complexes and nanoparticles against bacteria has not been assessed yet and could be an interesting approach for utilizing lower azole concentrations for antibacterial activity. In this work, we report the synthesis and characterization of new air-stable cobalt(II) and copper(II) complex derivatives of azoles and their antibacterial activities alone and in combination with AgNPs, which resulted in increased activity.

## 2. Discussion

**Scheme 1:**
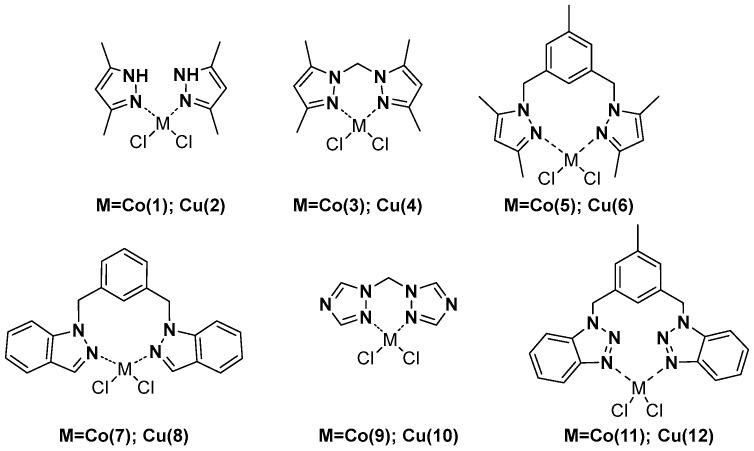
Azole complexes.

Complexes **1**–**4** and **9** and **10** (Scheme 1) have been reported previously (see the Materials and Methods and Supporting Information). The key point in the synthesis of new complexes **5**–**8**, **11** and **12** was the choice of a suitable solvent for each reaction. The reactions were carried out in solvents that dissolved both the ligand and the MCl_2_ but did not solubilize the final complex. In the synthesis of **5**, **7** and **11**, various solvents were tested, i.e., ethanol, methanol (MeOH), acetone, tetrahydrofuran (THF) and acetonitrile (CH_3_CN), which dissolved the starting reactants. However, no reaction was observed immediately. The cobalt complexes were isolated as air-stable blue, non-hygroscopic solids. The syntheses of the copper complexes were carried out under mild conditions with yields of 44–91%.

### 2.1. Infrared Spectroscopy

The new complexes were analyzed by infrared spectroscopy, particularly looking for the band shifts due to metal coordination. Table 1 provides an overview of the main bands in the infrared spectra of the complexes. The bands were assigned considering previous reports of the ligands and similar complexes [8]. In complexes (**1** and **2**), the presence of the bands at 3344 cm^−1^ and 3265 cm^−1^, which are assigned to N-H vibrations, confirmed the coordination of the metals to the N2 of 3,5-dimethylpyrazole. We found a shift to higher wavenumbers with respect to the free ligand [8], indicating the rigidity of the molecule when the metal was present. In (**3** and **4**), the ring vibrations of (C=N) and (C-C)Pyr in the free ligand (1035 cm^−1^ and 970 cm^−1^, respectively) also showed the rigidity of the structure of the complex. This case was not present in complexes (**5**–**8**), where the ring vibrations were not affected by coordination likely because the ligand was too big, and the central rings were not influenced by the electron density of the metal. Variations in the infrared spectra of each complex were observed, showing the electronic differences of the corresponding metals. Additionally, it was possible to find M-Cl vibrations with very low intensities.

### 2.2. UV-Vis Spectroscopy

The UV-Vis spectra were recorded to study the electronic properties of the complexes and to relate the properties to possible structures. Most of the complexes obtained showed intense absorption bands (200–300 nm). In complex **9**, bands below 300 nm were not observed because the solvent showed a high absorbance in this zone. Such bands are attributed to metal to ligand charge transfer, and intra ligand π-π* transitions [13]. The cobalt complexes showed a blue color in the solid state. When dissolved in DMSO or CH_3_CN, this color remained, while if they were dissolved in MeOH (complex **7**) the color changed to pale pink, a characteristic color of octahedral cobalt complexes with coordinated water. Complexes **1**, **9** and **11** showed bands at 600–700 nm, corresponding to ^4^*A*_2g_ ← ^4^*T*_2g_ and ^4^*A*_2g_ ← ^4^*T*_1g_ [14].

Complexes **3** and **5** showed a third band in the visible region with higher energy. This band is related to an equilibrium between tetrahedral and octahedral geometries given by coordination with solvents H_2_O or CH_3_CN. In complex **7**, a band at 525 nm (ε = 17 M^−1^ cm^−1^) was observed, which is consistent with Co(II) octahedral complexes [14].

For the Cu(II) complexes, charge-transfer bands were observed with high intensity in the UV region of the spectra that could be related with transitions of the organic moiety in the complex and to charge transfer from the nonbonding orbital of chloride to the vacant copper(II) d orbitals [15].

Additionally, overlapped signals were found due to interelectronic repulsion. In the case of complexes **4** and **6**, it is possible to assign the signals near 300 nm to charge transfer between chloride and copper. In the visible region near 500 nm, the electronic transition of *t*_2g_ ← *e*_g_ was found, which is characteristic for *d*^9^ metals [16].

### 2.3. Theoretical Calculations

Despite several attempts, no crystals suitable for X-ray diffraction analysis were obtained. Further computational studies were carried out for the new complexes to support the characterized structures. The calculated data are given on supporting information, which agrees well to the discussed main bands in the FTIR and UV-Vis spectra and their assignation (see above). For **5**–**8**, **11** and **12**, a doublet ground state was obtained (i.e., one unpaired electron).

All the complexes exhibit a slightly distorted tetrahedral geometry. Thus, all the series can be ascribed as tetrahedral complexes of Co(II) and Cu(II). Mostly of the upaired electron resides in the -CoCl_2_ and -CuCl_2_ fragment according to the obtained spin-denisty. The resulting structures support the expected systems according to the employed ligand. Such structures, exhibits isostructural species between Co(II) and Cu(II), which can be viewed as a useful template for further evaluation of relationship between different metal centers and molecular properties.

**Scheme 2:**
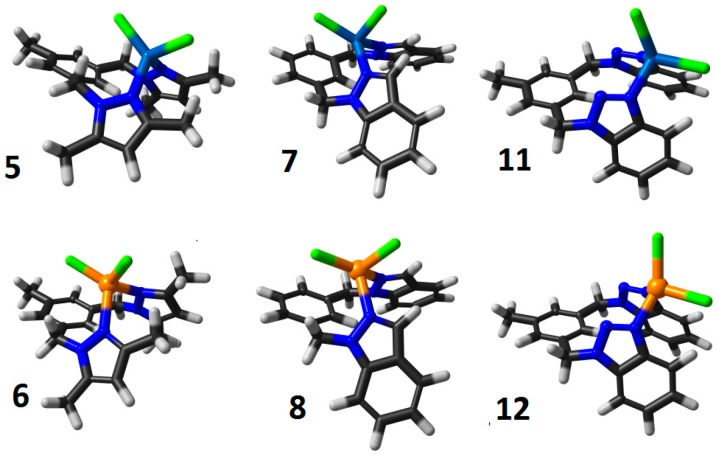
Optimized geometries for **5**–**8**, **11** and **12**, from computational calculations in agreement to Scheme 1.

Moreover, the location of the -MCl_2_ fragment in relation to the central phenyl ring is shifted between **5**–**8** and **11**, **12**, which can be a useful ability to design further selective catalytic species (Scheme 2).

### 2.4. Thermal Analysis

Thermogravimetric analyses were performed to study the different mass losses and to relate these losses to possible structures. The mass losses are based only on the percentages observed in the thermograms because the study was carried out without a detection system (TGA-MS). Complex **1** showed high stability because a residue at the end of the analysis was observed (pyrazole-Co). Complex (**2**) showed low thermal stability with 75% loss of the metal and 40% total loss of mass at 600 °C. In **3** and **4** rapid thermal degradations were observed in a few steps, indicating that stable intermediates were generated. Complexes **5** and **6** lost nearly 70% of the total mass at the end of the analysis, and complex **5** showed a very small loss (1.6%) at 150 °C, which could be related to the loss of water molecules coordinated to cobalt. However, the IR spectrum did not show bands related to H_2_O, so we assume that the mass loss corresponds to HCl. For complex **6**, the total loss of the ligand was observed at 700 °C. Complexes **7** and **8** exhibited good thermal stability with retention of almost half of the initial mass at 700 °C with observed losses of the indazole ring and 2HCl. Four mass losses were observed for complex **8**, which were analyzed in three different steps: first, a loss of 2HCl; then, a loss of benzene; and finally, decomposition of one indazole group. The mass losses of complex **9** were associated with the splittings of the CH_2_ spacers and triazoles.

### 2.5. Characterization of AgNPs

#### 2.5.1. Atomic Force Microscopy (AFM)

**Figure 1:**
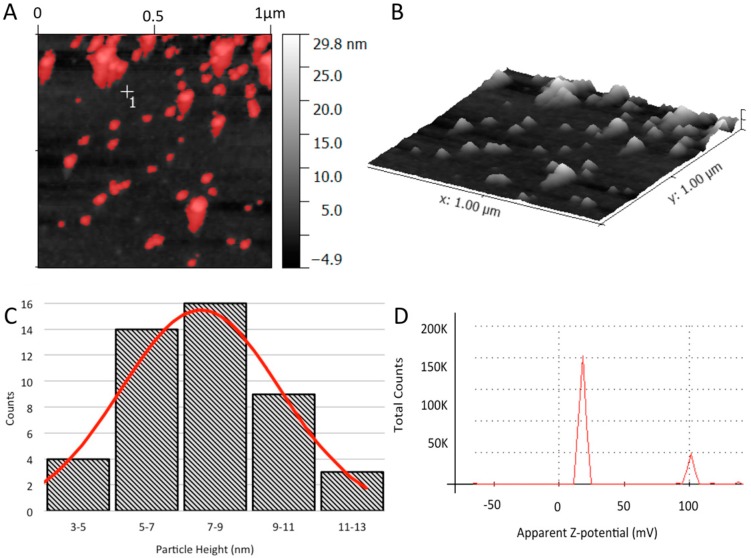
AgNPs characterization. (**A**) AFM image of the nanoparticles dispersed on silica glass; (**B**) 3D reconstruction of the AFM image; (**C**) Size distribution of the nanoparticles by height; (**D**) Z-potential measurements using DLS. These nanoparticles exhibited a small positively charged potential of approximately 18 ± 3.6 mV. There was also a secondary peak at 100 mV corresponding to the free PVP coating.

The nanoparticle size, size distribution and shape were measured using AFM. The AgNPs were coated with polyvinylpyrrolidone (PVP) and dried without sonication to mimic the methodology in the culture plates. Our results show spherical nanoparticles (Figure 1A,B). The AgNPs agglomerated on clusters up to 50 nm, due to the surface charge of the nanoparticles. Nevertheless, the average height of the single nanoparticles was 8.35 nm, with a height distribution between 5 to 13 nm (Figure 1B). These results suggest that the sized of the tested nanoparticles are in the range with the more effective antibacterial properties according with the literature [17,18].

#### 2.5.2. Dynamic Light Scattering (DLS)

We calculated the Z-potential (δ) from the electrophoretic mobility measurements and the Henry equation as shown in Equation (1):

(1) $$U_{E}=\frac{2\varepsilon \zeta \;f\left(ka\right)}{3\eta }$$

where $U_{E}$ is the electrophoretic mobility, ζ is the z-potential, ε dielectric constant, f(ka) is the Henry’s function and η is the solution viscosity [19]. Our Z-potential results evidenced that the AgNPs had positively charged surfaces of 18 ± 3.6 mV in water, as shown in Figure 1D. The Z-potential is correlated to the impact of the bio-interactions of the nanoparticles, specifically with cellular uptake and protein adsorption. Nanoparticles with a positively charged surface are more susceptible to conformations of the protein corona and could be more easily taken in by cells [20]. Positively charged particles have been reported to exert antimicrobial effects by adhering to Gram-negative bacteria [21]. Therefore, we expected more of an antibacterial effect from the 10-nm AgNPs on Gram-negative strains than on Gram-positive strains.

### 2.6. Biological Activity

The biological activity of the AgNPs alone, azole complexes without AgNPs and the azole complexes combined with AgNPs were evaluated in triplicate against eight bacterial strains, MRSA, *S. typhimurium*, *E. aerogenes*, *B. cereus*, *E. faecalis*, *S. flexneri*, *S. aureus* and *E. coli*, after 8 h and 16 h of exposure.

#### 2.6.1. Antibacterial Activity of the AgNPs Only

**Figure 2:**
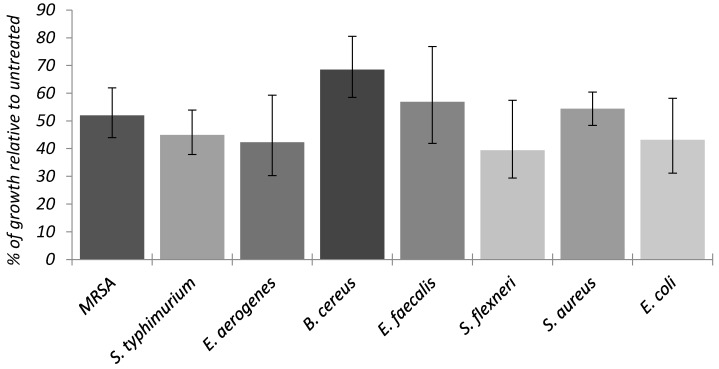
Antibacterial effects of AgNPs alone on four Gram (+) and four Gram (−) strains. The AgNPs’s size were 10 nm at 2 µg mL^−1^.

The antibacterial activities of AgNPs have been reported, and it has been shown that their size, and concentration define their effectiveness. Nanoparticles with sizes from 5 to 100 nm at a concentration of 40 μg mL^−1^ have been reported to have bactericidal effects on most tested strains [22]. Sizes under 30 nm are more active than bigger nanoparticles, and it is thought that the activity is associated with an easier release of silver ions from the smaller particles [17]. At the same time, particles with spherical shapes show better results, almost five times more effective than particles with different shapes [22,23]. Our experiments were limited to spherical AgNPs with sizes of 10 nm at 2 μg mL^−1^ concentration. Additional experiment with nanoparticles sizes between 30 and 100 nm did not show bactericidal effects, data not showed. The concentration of 2 μg mL^−1^ was used to evaluate the synergistic effect with the azole complexes, since the literature reports only bacteriostatic effects at this concentration [2,22]. Therefore, any bactericidal effects depend on the AgNP-Azole complex synergy exclusively. In fact, all the strains in our experiments showed bacteriostatic effects at this concentration (Figure 2).

Bacteria were able to keep growing even in the presence of 2 µg mL^−1^ AgNPs. However, at the same time, none of the strains were able to grow at 100% compared to the untreated control. Some bacteria were more sensitive to the AgNPs, such as *E. aerogenes*, *S. typhimurium*, *S. flexneri* and *E. coli.* Interestingly, and in contrast with previous studies that reported that AgNPs had a major bactericide effect against gram-negative bacteria [21], we found that the AgNPs showed similar activity against both Gram-positive and Gram-negative bacteria strains. This is most likely because Ag^+^ ions react strongly with biological substances, such as proteins, enzymes, DNA, and RNA, due to the interactions that occur with thiol, carboxylate, phosphate, hydroxyl, imidazole, indole or amine functional groups. These interactions can be produced simply or in combination, which can lead to a series of events that interfere with vital microbial processes [24]. It has also been reported that silver reacts with the sulfhydryl (-S-H) groups on cell walls to form R-S-S-R bonds, thus, blocking respiration and causing cell death [17].

#### 2.6.2. Antibacterial Activity of the Azole Complexes with and without AgNPs

The antibacterial activities of the ligands, its complexes and metal complexes combined with AgNPs against the bacterial strains were also studied. The free ligands showed no activity against any bacterium. This may be because azoles have higher anti-fungal activities since they inhibit the ergosterol synthesis in fungal cell walls [25,26]. However, some azole-derivative compounds have exhibited moderate antimicrobial activities [27,28,29,30], and it has been found that azoles in bacteria are inhibitors for enoyl acyl carrier protein reductase [31]. Manganese tricarbonyl coordinated to ketoconazole, miconazole, or clotrimazole showed higher antibacterial activity compared to the free azole only for Gram-positive bacteria [32].

We studied the antibacterial activities of twelve complexes derived from azole, which contained cobalt and copper. In previous studies carried out in our research group, it was found that metal complexes containing copper and cobalt instead of zinc displayed better antibacterial effects against bacterial strains [8]. The in vitro antibacterial results of complexes **3**–**5**, **8** and **9** with and without AgNPs are summarized in Table 2 and Table 3.

As observed in Table 2 and Table 3, complexes **3**–**5**, **8** and **9** showed high antibacterial activities. The results suggest that these compounds at 8 and 16 h were effective against at least one of the strains tested in this study. This finding is likely related to the better solubility, bioavailability and lipophilicity of the complexes that reduced the permeability barriers of the cells and slowed the normal cellular processes of the microorganisms, resulting in increased antimicrobial activities or chelating effects [8,31,32,33]. At 16 h, better sensitivities to the bacteria for all the azole complexes were observed (Table 3). However, the doses required to generate bactericidal effects were increased for complexes **5** (*S. aureus* and MRSA) and **3** (MRSA and *E. faecalis*), which is one of the bacterium that is indicated by the CDC as a serious threat (Table 2 and Table 3). The complexes inhibited higher activities against Gram-negative strains than Gram-positive strains. If we compare complexes **3** and **4**, where only the metal center differs in their structures, complex **3** showed higher antimicrobial activity. This might be attributed to the presence of cobalt. Cobalt has been reported to be a more toxic element than copper [34]. Co has a larger atomic radius than Cu, and it has been reported that the bond length between Co and its ligand could favor antibacterial activity [35]. In addition, copper is an essential trace element for many biological processes [36]. As the data analysis further indicates, complexes **5**, **8** and **9** showed more moderate activities than **3** and **4**. It appears that the presence of the pyrazole ring favors antibacterial activity compared to complexes containing indazole or triazole. Some studies have shown that pyrazoles in complexes can inhibit DNA gyrase and topoisomerase IV in bacteria but are more effective against Gram-positive bacteria than Gram-negative bacteria [37]. The binding of specific metal ions to azoles contributes to the antibacterial activity.

In addition, the combination of AgNPs with the metal complexes showed bactericidal effects at lower concentrations than in the absence of AgNPs for both Gram-positive and Gram-negative bacteria strains. The combination of metal complexes with AgNPs showed bactericidal activities while the free complexes and AgNPs alone just had bacteriostatic effects.

When complex **5** was combined with AgNPs, a 10-fold increase in antimicrobial activity was observed for *S. aureus* (Table 2), whereas in the case of *S. flexneri*, *E aerogenes* and *S. typhymurium*, we only observed a 2-fold increase in activity. Interestingly, this complex showed better biological results against Gram-negative bacteria, probably due to the presence of toluene, which is known to induce increased fluidity in Gram-negative cell membranes [8].

Complex **8** in the presence of AgNPs showed 3-fold increased activities against *S. aureus* and *E. aerogenes* (Table 2). Likewise, in the case of **4** with AgNPs, 2-fold increased activities against *S. flexneri* and *E. faecalis* were evidenced. Compounds **3** and **9** showed synergistic antibacterial effects but in minor proportions. These results may be due to the fusion of two antimicrobial agents with distinct modes of action, the chelating effect of the complexes and the presence of Ag^+^ ions (AgNPs), that react with DNA and enzymes of bacteria. To the best of our knowledge, the antibacterial activities of AgNPs combined with azole complexes have not been previously reported. Additionally, the antibacterial activity of AgNPs (10 nm) at a concentration of 2 μg mL^−1^ has not been reported.

### 2.7. Physicochemical Characterization of the Colloids

**Figure 3:**
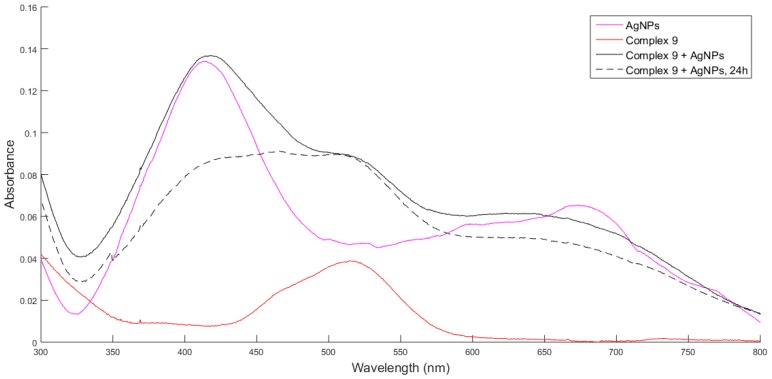
UV/Vis spectra to show the interaction between complex **9** and AgNPs.

In Figure 3, it is can be seen that AgNPs have their highest absorbance between 417 nm and 678 nm, while complex **9** has a maximum absorbance at 516 nm.

The spectrum of the solution with complex **9** and the AgNPs seems to be the sum of the absorbance of each component, with a slightly change in 650–750 nm range. However, when the spectra was recorded 24 h later, the absorption due to nanoparticles decreased significantly, probably because the complex molecules were adsorbed over the surfaces of the AgNPs. Additionally, there is no change in the absorbance wavelength of the components, suggesting that there is no change in nanoparticle size and that the metal centers of the complexes have no effect on absorbance. These suggest that the interaction between the azol complexes and AgNPs are just physical, without a change in the structure of the coordination compound or the nanoparticles.

The results suggest that the presence of AgNPs could effectively improve the antibacterial activities of some azole complexes exhibiting broad-spectrum biocidal activities toward many different microbial strains. The antibacterial efficacies could be related to the azolyl ring, the metal center and the AgNPs diffusion, but further studies are necessary to elucidate the antibacterial mechanism with combination of AgNPs with metal complexes.

### 2.8. Cytotoxicity

**Figure 4:**
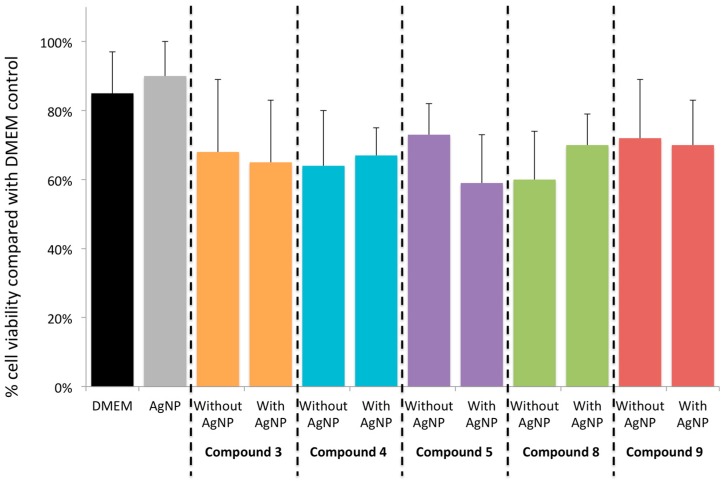
Cytotoxic effects of azole complexes (50 μg mL^−1^) alone and in combination with AgNPs (10-nm diameter at 2 µg mL^−1^). The HFF cells were exposed to the complexes and AgNPs for 24 h.

Mammalian cells exposed to AgNPs alone did not evidence signs of cytotoxic effects. The viability assays using 3-(4,5-dimethylthiazol-2-yl)-2,5-diphenyltetrazolium bromide (MTT) reported viabilities higher than 90%. However, the combination of most azole complexes with AgNPs had a deleterious effect, except for compounds **4** and **8** (Figure 4).

The fibroblast viability was always higher than 60% for all complexes in combination with AgNPs; these values suggest a toxic effect at 50 µg mL^−1^. Although lower doses could be employed in animal studies without losing antibacterial effect, these studies are required to identify the potential LD_50_ of the compounds with AgNPs [17].

## 3. Materials and Methods

### 3.1. General Information

All manipulations were routinely performed in an inert atmosphere (nitrogen) using standard Schlenk-tube techniques. All reagent-grade solvents were dried, distilled, and stored under a nitrogen atmosphere. The starting compounds were prepared based on the literature: bis(3,5-dimethyl-1-pyrazolyl)methane [38], bis(1,2,4-triazol-1-yl)methane [39], 3,5-bis(3,5-dimethylpyrazol-1-ylmethyl)toluene [40], 3,5-bis(benzotriazol-1-ylmethyl)toluene [41] and 1,3-bis(indazol-1-ylmethyl)benzene (L). The synthetic protocols for the previously reported complexes dichloro[bis(3,5-dimethylpirazol-NN)]cobalt(II) (**1**), dichloro[bis(3,5-dimethylpirazol-NN)]-copper(II) (**2**), dichloro[bis(3,5-dimethyl-1-pyrazolyl)methane-NN]cobalt(II) (**3**), dichloro[bis(3,5-dimethyl-1-pyrazolyl)methane-NN]copper(II) (**4**), dichloro[bis(1,2,4-triazol-1-yl)methane-NN]-cobalt(II) (**9**) and dichloro[bis(1,2,4-triazol-1-yl) methane-NN]copper(II) (**10**) are provided in the Supplementary Information [8]. Elemental analyses (C, H and N) were performed with a FLASH 2000 CHNS/O Analyzer (Thermo Fisher Scientific, Waltham, MA, USA). Fourier-transform infrared (FTIR) spectra were recorded on a Thermo Nicolet NEXUS FTIR spectrophotometer (Thermo Fisher Scientific) using KBr pellets Melting points were determined on a Mel-Temp^®^ 1101D apparatus (Eletrothermal, Staffordshire, UK) in open capillary tubes, and they are reported uncorrected. Ultraviolet-visible (UV-Vis) spectra were recorded on a Cary 100 spectrophotometer (Agilent Technologies, Kansas City, KS, USA). Thermogravimetric analyses (TGA) of the complexes were obtained on a NETZSCH STA 409 PC/PG from 8 to 10 mg of the complexes in nitrogen media. The samples were subjected to dynamic heating over a temperature range of 30–700 °C with a heating rate of 10 °C min^−1^. The TG curves were analyzed to give the percentage mass loss as a function of the temperature.

### 3.2. Synthesis of the Complexes

#### 3.2.1. Dichloro[3,5-bis(3,5-dimethylpyrazol-1-ylmethyl)toluene-NN]cobalt(II) (**5**)

A solution of 3,5-bis(3,5-dimethylpyrazol-1-ylmethyl)toluene (0.33 mmol; 101,4 mg) in acetonitrile (CH_3_CN, 2 mL) was added to a solution of CoCl_2_ (0.38 mmol; 49.8 mg) in CH_3_CN (5 mL). The reaction mixture was refluxed for 12 h and dried under vacuum. A pure blue compound was obtained by crystallization from dichloromethane (DCM). Yield: 128 mg (89%). M.p.: 192–193 °C. IR (KBr) ν/cm^−1^: 2921m, 1553s, 1468s, 1421s, 1368s, 1271w, 1047m, 799m, 729s. Anal. calc. for C_19_H_24_N_4_CoCl_2_: C, 52.07; H, 5.52; N, 12.78%. Found C, 51.87; H, 5.51; N, 12.72%. UV/Vis (CH_3_CN): λ_max_/nm (ε/(L·mol^−1^·cm^−1^)) = 225 (2666), 570 (301), 616 (328), 678 (441).

#### 3.2.2. Dichloro[3,5-bis(3,5-dimethylpyrazol-1-ylmethyl)toluene-NN]copper(II) (**6**)

A solution of 3,5-bis(3,5-dimethylpyrazol-1-ylmethyl)toluene (0.65 mmol; 200.5 mg) in acetone (3 mL) was added to a solution of CuCl_2_ (0.65 mmol; 87.4 mg) in acetone (5 mL). The reaction mixture was stirred at r.t. for 12 h. A green solid formed and was filtered off, washed with acetone and dried under vacuum. Yield: 251 mg (87.4%). M.p.: 185–186 °C. IR (KBr) ν/cm^−1^: 3445w, 2926m, 1608m, 1556vs, 1469s, 1424s, 1385s, 1302m, 1055m, 843m, 799m, 786m, 470w. Anal. calc. for C_19_H_24_N_4_CuCl_2_: C, 51.53; H, 5.46; N, 12.65%. Found C, 51.22; H, 5.25; N, 11.87%. UV/Vis (CH_3_CN): λ_max_/nm (ε/(L·mol^−1^·cm^−1^)) = 219 (19,666), 275 (2974), 355 (1369), 454 (459).

#### 3.2.3. Dichloro[1,3-bis(indazol-1-ylmethyl)benzene-NN]cobalt(II) (**7**)

A solution of 1,3-bis(indazol-1-ylmethyl)benzene (0.29 mmol; 99.6 mg) in CH_3_CN (3 mL) was added to a solution of CoCl_2_ (0.30 mmol; 38.5 mg) in CH_3_CN (3 mL). The reaction mixture was refluxed for 3 h. A blue solid formed and was filtered off, washed with CH_3_CN and diethyl ether and dried under vacuum. Yield: 91.3 mg (66.2%). M.p.: 328 °C. IR (KBr) ν/cm^−1^: 3094m, 1628m, 1519m, 1438m, 1310m, 1150m, 1030w, 852m, 758vs, 740m, 435w. Anal. calc. for C_22_H_18_N_4_CoCl_2_: C, 56.43; H, 3.87; N, 11.97%. Found C, 56.19; H, 3.82; N, 11.02%. UV/Vis (MeOH): λ_max_/nm (ε/(L·mol^−1^·cm^−1^)) = 275 (17,343), 294 (14,548), 525 (17).

#### 3.2.4. Dichloro[1,3-bis(indazol-1-ylmethyl)benzene-NN]copper(II) (**8**)

A solution of 1,3-bis(indazol-1-ylmethyl)benzene (0.59 mmol; 199.0 mg) in acetone (5 mL) was added to a solution of CuCl_2_ (0.60 mmol; 80.7 mg) in acetone (5 mL). The reaction mixture was stirred at r.t. for 12 h. A red solid formed and was filtered off, washed with acetone and dried at 80 °C for 6 h. Yield: 183 mg (65.7%). M.p.: 205 °C. IR (KBr) ν/cm^−1^: 3446w, 3098m, 1628m, 1520s, 1431m, 1375m, 1152s, 914m, 755vs, 615w, 432w. Anal. calc. for C_22_H_18_N_4_CuCl_2_: C, 55.82; H, 3.84; N, 11.85%. Found C, 55.80; H, 3.64; N, 11.71%. UV/Vis (MeOH): λ_max_/nm (ε/(L·mol^−1^·cm^−1^)) = 275 (18,782), 293 (16,064).

#### 3.2.5. Dichloro[3,5-bis(benzotriazol-1-ylmethyl)toluene-NN]cobalt(II) (**11**)

A solution of 3,5-bis(benzotriazol-1-ylmethyl)toluene (0.28 mmol; 99.5 mg) in THF (10 mL) was added to a solution of CoCl_2_ (0.31 mmol; 40.8 mg) in THF (4 mL). The reaction mixture was refluxed for 20 h. A blue solid formed and was filtered off, washed with THF and diethyl ether and dried at 80 °C for 12 h. Yield: 111.9 mg (82.3%). M.p.: 212–215 °C. IR (KBr) ν/cm^−1^: 3092w, 1610m, 1456m, 1318m, 1229m, 1169w, 749s, 434w. Anal. calc. for C_21_H_18_N_6_CoCl_2_: C, 52.09; H, 3.75; N, 17.36%. Found C, 52.08; H, 3.66; N, 17.32%. UV/Vis (DMSO): λ_max_/nm (ε/(L·mol^−1^·cm^−1^)) = 263 (8615), 283 (7862), 614 (97), 679 (161).

#### 3.2.6. Dichloro[3,5-bis(benzotriazol-1-ylmethyl)toluene-NN]copper(II) (**12**)

A solution of 3,5-bis(benzotriazol-1-ylmethyl)toluene (0.28 mmol; 99.5 mg) in acetone (5 mL) was added to a solution of CuCl_2_ (0.28 mmol; 37.5 mg) in acetone (5 mL). The reaction mixture was stirred at r.t. for 5 h. A green solid formed and was filtered off, washed with acetone and dried under vacuum. Yield: 116 mg (85.1%). M.p.: 236–237 °C. IR (KBr) ν/cm^−1^: 3131w, 2358w, 2343w, 1609w, 1457m, 1233s, 746vs, 669w, 432w. Anal. calc. for C_21_H_18_N_6_CuCl_2_: C, 51.60; H, 3.71; N, 17.19%. Found C, 51.24; H, 3.66; N, 17.15%. UV/Vis (DMSO): λ_max_/nm (ε/(L·mol^−1^·cm^−1^)) = 263 (12,769), 284 (9667).

### 3.3. Characterization of AgNPs

Commercial silver nanoparticles with an average diameter of 10 nm (Cat. 796379 Sigma Aldrich, Waltham, MA, USA) were characterized by dynamic light scattering (DLS) and atomic force microscopy (AFM). The hydrodynamic size distribution and zeta potential of the AgNPs were measured in water and solution media by DLS using a Zetasizer Nano ZS system (Model ZEN3600, Malvern Instruments Ltda., Malvern, UK). A suspension of AgNPs were sonicated prior to each measurement. A tip sonicator (Cat. Q700 QSonica, Newtown, CT, USA) was used with a sequence of 20 s of sonication and 40 s of rest over 15 min. The sonicated samples were placed into disposable cuvettes (Cat. #DTS1070, Malvern Instruments). Each sample of AgNPs were measured ten times at room temperature. The refractive index used was 0.056 at a wavelength of 633 nm, and the absorption coefficient was 8.49 × 10^−5^ cm^−1^ [42].

#### 3.3.1. Atomic Force Microscopy (AFM)

The size distributions and shapes of the particles were measured using an atomic force microscope (Asylum Research MFP-3D Bio, Santa Barbara, CA, USA) with a cantilever operated in tapping mode for image topography. The cantilever was silicon nitride (Si_3_N_4_) with a nominal force constant of 0.06 N/m. The scan rate was set to 1 Hz on a scanned area of 2 µm. The samples were imaged at a preset constant force of interaction of the order of 5 nN. The adhesion forces between the tips and the samples, at fixed locations, were determined at the point of disengagement of the tip in the force (F) versus separation (Z) curves (withdraw). Here, the tip experiences a pull-off force F = kδ, where δ is the deformation at disengagement and k is the cantilever force constant (ten samples were measured).

#### 3.3.2. Physicochemical Characterization of the Colloids

The solutions were prepared in DMSO-Water (1:1) to obtain a concentration equal to 5 mM of complex **9** and 0.005 mg/mL of AgNPs.

### 3.4. In Vitro Antibacterial Activity

#### 3.4.1. Reagents

The solvents used were of analytical grades, and all metal(II) precursors were used as chloride salts. Silver nanoparticles (10 nm (795941) and 30 nm (795925)) were used as controls and were obtained from Sigma-Aldrich. The 20-nm particles were purchased from US Research Nanomaterials (US1037, Houston, TX, USA) and prepared following the manufacturer’s instructions at 100 µg mL^−1^. The metal(II) salts, ligands and metal complexes were prepared as 200 mM solutions in DMSO or dimethylformamide (DMF). Complex **3**–**5** and **9** were dissolved in DMSO at 0.6%, 0.6%, 0.5% and 0.7% respectively. Complex **8** was dissolved in DMF at 0.4%.

#### 3.4.2. Strains and Isolates

All metal salts, ligands, metal complexes, AgNPs and AgNPs-complexes were screened in vitro for their antibacterial activities against four Gram-positive (*Staphylococcus aureus* (ATCC 22923), Methicillin-resistant *Staphylococcus aureus* (MRSA), *Enterococcus faecalis* (ATCC 19433) and *Bacillus cereus* (ATCC 14579)) and four Gram-negative (*Escherichia coli* (ATCC 25922), *Enterobacter aerogenes* (ATCC 13048), *Salmonella typhimurium* (ATCC 14028) and *Shigella flexneri* (ATCC 29903)) bacterial strains. All strains used were obtained from the American Type Culture Collection (ATCC, Manassas, VA, USA) and stored as glycerol stocks in the Laboratory of Mycology and Phytopathology, at Universidad de los Andes, Bogotá D.C.

#### 3.4.3. Antibacterial Activities

For every experiment, each of the bacterial strains were freshly grown from the stored stock in Luria broth (LB)-agar plates (Oxoid, Hampshire, UK) for 24 h at 30 °C. An inoculum from these plates was used to grow cultures in 5 mL of LB-media for 24 h at 250 rpm at 37 °C. For screening, these bacterial cultures were seeded again in fresh LB-media (1:12,000) for ~3 h until a concentration of 10^5^ CFU mL^−1^ (0.4–0.5 OD_600nm_) was obtained on a shaker-incubator. A total of 90 μL of pre-inoculated aliquots (OD_600nm_ of 0.001) were placed into 96-well clear flat-bottom plates (Corning-Costar Corp, Corning, NY, USA) with final volumes of 100 μL. Bacterial growth kinetics were measured in the 96-well plates at 37 °C without shaking for 8 and 16 h. Bacteria strains were exposed in parallel for 8 and 16 h to the complex alone, AgNP alone and, AgNP + Complex independently. The screening of the metal salts, ligands and metal complexes were tested at 500 µg mL^−1^ in triplicate, and the experiments were repeated twice. AgNPs and AgNPs-complexes were screened at three different concentrations (2 μg mL^−1^, 1 μg mL^−1^ and 0.5 μg mL^−1^) maintaining the metal salts, ligands, metal complexes at 500 µg mL^−1^. The compounds used as controls included the antibiotics gentamicin and kanamycin (Roche Diagnostics, Indianapolis, IN, USA), and these were tested at final concentrations of 20 μg mL^−1^ or 2 μg mL^−1^. All analyses were performed using the GraphPad Prism version 7.02 (GraphPad Software, La Jolla, CA, USA).

#### 3.4.4. Mammalian-Cell Cytotoxicity Assay

Primary neonatal human foreskin fibroblasts (HFF) were maintained in Dulbeco’s modified Eagle’s medium (DMEM) (Sigma, Waltham, MA, USA) supplemented with 5% or 10% fetal bovine serum (FBS) (Gibco, Waltham, MA, USA), 1% penicillin/streptomycin (Gibco) and 2% glutamine (Gibco). The HFF cells were grown as a monolayer in Roswell Park Memorial Institute (RPMI) 1640 medium (Sigma) supplemented with 10% FBS (Gibco), 1% penicillin/streptomycin (Gibco) and 2% glutamine (Gibco). The cultures were incubated at 37 °C in a humidified 5% CO_2_ atmosphere during two complete cell cycles (24 h) before treatment.

The cytotoxicity was determined by a colorimetric method based on the reduction of 3-[4,5-dimethylthiazol-2-yl]-2,5-diphenyltetrazolium bromide by viable cells (MTT) described by Mosmann [43]. Briefly, in a 96-well flat-bottom plate, 3 × 10^5^ HFF cells per well were grown (90% confluence), with four replicate wells for each treatment and were washed twice in PBS and resuspended in DMEM without FBS. Cells were exposed to two different concentrations of the metal salts, ligands, metal complexes at 500 μg mL^−1^ or 50 μg mL^−1^ at a fixed concentration of 2 μg mL^−1^ for the 10-nm nanoparticles. Cells treated with 10% DMSO were the positive control, and untreated cells (1% DMSO) served as the negative control. The plate was then incubated at 37 °C in a humidified 5% CO_2_ atmosphere for 24 h. After incubation time, 10 µL of MTT (5 mg mL^−1^ phosphate buffered saline solution) was added to each well, and the cells were incubated for a further 4 h. To dissolve the formazan crystal, 100 µL of DMSO were added to each well. Formazan crystals were dissolved by agitation at 37 °C. After 5 min, each well was analyzed in a microplate reader (BioRad, Hercules, CA, USA) at 595 nm using a reference wavelength of 655 nm. The percentage of viable treated cells was calculated in relation to the untreated controls (viability percentage = OD-treated cells/OD control cells × 100%). The untreated control was taken as 100%.

#### 3.4.5. Growth Inhibition of the Bacteria Strains

Growth inhibition assays were performed as previously described with a slight modification. Briefly, a culture (5 mL) of bacteria strains were grown from a single colony for 8 h at 37 °C in Lysogeny Broth (LB) media and diluted back to an OD_600_ of 0.001. A 96-well plate assay was set up with 90 μL of the respective cultures in LB-media either with DMSO or containing 10–100 μg mL^−1^ of the compounds. The experiment was repeated with and without 2 μg mL^−1^ of the 10-nm nanoparticles. The OD_600_ for all wells was recorded on a Multiskan™ GO microplate spectrophotometer (Thermo Scientific™, Waltham, MA, USA) at 0, 8, 16 and 32 h. To assure there were no absorbance contributions from the inhibitors, a change in OD_600_ was determined by the subtraction of the absorbance from the blank containing an equivalent concentration of inhibitor. The concentration of the compound that inhibited 50% of the microorganism growth was calculated using the GraphPad Prism version 7.02.

## 4. Conclusions

In summary, we have synthesized and characterized a series of air-stable cobalt and copper complexes with azole ligand derivatives. Experimental and computational studies predict that all the complexes exhibit a slightly distorted tetrahedral geometry. In vitro assays showed that complexes **3**–**5**, **8** and **9** exhibited moderate antibacterial activities. However, their antibacterial activities increased when combined with AgNPs at a constant concentration of 2 µg mL^−1^, which also impacted the reduction of the azole concentration up to 10 times. The synergistic effect of the azoles and AgNPs did not impact the HFF cellular viability. The complexes combined with AgNPs showed promising activities against different bacterial strains. Further studies to acquire more information concerning their structure-activity relationships are in progress.

## Figures and Tables

**Table 1:** Main bands in the FTIR spectra for the complexes. Pyr = pyrazole; Ind = indazole; Tol = toluene; vs = very strong; s = strong; m = medium; w = weak; vw = very weak.

Compound	Wavenumber ν (cm^−1^)				
	(N-H)	(C-H)	(C-H)_Pyr_	(C-N)	(M-Cl)
1	3344s	3142w	1568vs	1470m	427m
2	3265vs	3147vs	1570vs	1472m	431m
	(C-H)	(C-CH_3_)	(C=N)	(C-C)_Pyr_	(M-Cl)
3	3011w	1465s	1051m	1003m	493w
4	3027w	1467s	1044m	1001m	492w
	(C-H)	(C-CH_3_)	(C-C)_Pyr_	(C-C-N)	(M-Cl)
5	3127m	1468m	1608m	729m	419w
6	3137w	1469m	1608m	738m	420w
	(C-H)	(C-C)_Ind_	(C-N)_Ind_	(C-H)_Ind_	(M-Cl)
7	3094m	1628s	1519s	1478m	490w
8	3098m	1628s	1519s	1477m	497w
	(C-H)	(H_2_C-N)	(N-N)	(C-N)	(M-Cl)
9	3115w	1518s	1458m	1362w	507vw
10	3137s	1525s	1460m	1357w	503vw
	(C-H)	(C-C)_Tol_	(N-N)	(N=N)	(M-Cl)
11	2970w	1610m	1284m	1229s	419w
12	2968m	1610m	1288m	1233s	419w

**Table 2:** Minimum inhibitory concentrations (MIC) (**^¶^**) and minimum bactericidal concentrations (MBC, μg mL^−1^) of bacterial strains exposed to azole complexes and 10-nm AgNPs (2 μg mL^−1^) for 8 h.

Complex Bacteria Strain	3		4		5		8		9	
	Without AgNPs	With AgNPs *	Without AgNPs	With AgNPs *	Without AgNPs	With AgNPs *	Without AgNPs	With AgNPs *	Without AgNPs	With AgNPs *
*S. aureus* (+)	>500	>500	200 ^¶^	200	250	25	200	62.5	400 ^¶^	250 ^¶^
*S. flexneri* (−)	200	125	200	100	200	62.5	125	100	250	250
MRSA (+)	62.5	62.5	250	200	200 ^¶^	200	200	250	>500	N/A
*E. faecalis* (+)	62.5 ^¶^	100	400	200	250 ^¶^	250 ^¶^	400	400	>500	N/A
*E. aerogenes* (−)	>500	>500	>500	400	400	250	400	250	>500	500 ^¶^
*B. cereus* (+)	>500	>500	400	400	>500	>500	500 ^¶^	400 ^¶^	>500	>500
*E. coli* (−)	125	125	>500	500	400	400	500	500	>500	250
*S. typhymurium* (−)	250 ^¶^	250 ^¶^	N/A	250 ^¶^	400	250	400 ^¶^	400 ^¶^	N/A	500 ^¶^

* AgNPs with a 10-nm diameter were added at the same concentration of 2 μg mL^−1^; **^¶^** Bacteria exhibited only inhibitory activities.

**Table 3:** Minimum inhibitory concentrations (MIC) (**^¶^**) and minimum bactericidal concentrations (MBC, μg mL^−1^) of bacterial strains exposed to azole complexes and 10-nm AgNPs (2 μg mL^−1^) for 16 h.

Complex Bacteria Strain	3		4		5		8		9	
	Without AgNPs	With AgNPs *	Without AgNPs	With AgNPs *	Without AgNPs	With AgNPs *	Without AgNPs	With AgNPs *	Without AgNPs	With AgNPs *
*S. aureus* (+)	100	100	100	100	250	250	200	200	400 ^¶^	200 ^¶^
*S. flexneri* (−)	500	250	100	50	200	125	100	50	200	200
MRSA (+)	250	250	250	250	200	200	200	250	500 ^¶^	500 ^¶^
*E. faecalis* (+)	400	200	400	200	200	200	400	400	>500	>500
*E. aerogenes* (−)	200	200	>500	250	500	500	400	400	>500	>500
*B. cereus* (+)	400	400	>500	>500	>500	>500	>500	>500	>500	>500
*E. coli* (−)	>500	400	200	200	400	400	400	400	500 ^¶^	250
*S. typhymurium* (−)	125 ^¶^	125 ^¶^	>500	250	400	400	400	400	>500	>500

* AgNPs with a 10-nm diameter were added at the same concentration of 2 μg mL^−1^; **^¶^** Bacteria exhibited only inhibitory activities.

## Supplementary Materials

The supplementary materials are available online.

## Abbreviations

AgNPs	Silver nanoparticles
WHO	World Health Organization;
CDC	Communicable Disease Center
FDA	Food and Drug Administration
MRSA	Methicillin-resistant *Staphylococcus aureus*
*E. aerogenes*	*Enterobacter aerogenes*
*S. typhimurium*	*Salmonella typhimurium*
*S. aureus*	*Staphylococcus aureus*
*E. faecalis*	*Enterococcus faecalis*
*B. cereus*	*Bacillus cereus*
*E. coli*	*Escherichia coli*
*S. flexneri*	*Shigella flexneri*
MBC	Minimum Bactericidal Concentration
TGA	Thermogravimetric analysis
MIC_50_	Minimum inhibitory concentration 50
THF	Tetrahydrofuran
CH_3_CN	Acetonitrile
FTIR	Fourier-transform infrared spectroscopy
HCl	Hydrochloric acid
UV-Vis	Ultraviolet-visible spectroscopy
MeOH	Methanol
DMSO	Dimethylsulfoxide
AFM	Atomic force microscopy
PVP	Polyvinylpyrrolidone
MTT	(3-(4,5-Dimethylthiazol-2-yl)-2,5-diphenyltetrazolium bromide) tetrazolium
DLS	Dynamic Light Scattering
HHF	Human Foreskin Fibroblast
KBr	Potassium bromide
DCM	Dichloromethane
DMF	Dimethylformamide
