# Supplementary material for: Antibacterial Activities of Azole Complexes Combined with Silver Nanoparticles
Source: Molecules. 2018 Feb 8;23(2):361. doi: 10.3390/molecules23020361 (PMC6017516; doi:10.3390/molecules23020361)
Supplement: Supplementary file 1 [file molecules-23-00361-s001.pdf]

Supplementary Information

ANTIBACTERIAL ACTIVITIES OF AZOLE COMPLEXES COMBINED WITH SILVER NANOPARTICLES

Nestor J. Bello-Vieda,[1] Homero F. Pastrana,[2] Manuel Garavito,[3] Alba G. Ávila,[2] Adriana M. Celis,[3] Silvia Restrepo[3], Alvaro Muñoz-Castro[4] and John. J Hurtado[1]

[1]Departamento de Química, Grupo de Investigación en Química Inorgánica, Catálisis y Bioinorgánica, Universidad de Los Andes, Cra. 1 N° 18A-12, 111711 Bogotá D.C, Colombia

[2]Departamento de Ingeniería Eléctrica y Electrónica, Centro de Microelectrónica, Universidad de Los Andes, Bogotá D.C., Colombia

[3]Departamento de Ciencias Biológicas, Laboratorio de Micología y Fitopatología, Universidad de Los Andes, Bogotá D.C., Colombia

[4]Grupo de Química Inorgánica y Materiales Moleculares, Universidad Autonoma de Chile, El Llano Subercaseaux 2801, Santiago, Chile.

*Corresponding author.
E-mail address: jj.hurtado@uniandes.edu.co (J. Hurtado)

Synthesis of complexes

dichloro[bis(3,5-dimethylpirazol-NN)]cobalt(II) (**1**)

This complex was prepared by using a modified procedure from the literature [1]. A solution of 3,5-dimethylpyrazol (7.70 mmol; 740.5 mg) in 5 mL of tetrahydrofuran (THF) was added to a suspension of $CoCl_2$ (3.87 mmol; 501.9 mg) in 15 mL of THF. The reaction mixture was refluxed for 4 h. The resulting mixture was filtered off and evaporated to dryness to give a dark blue solid which was re-crystallized with ethanol. Yield: 438.5 mg (44 %). M.p: 222-223°C. IR (KBr) ν/cm^{-1}: 3344vs, 3312vs, 2923w, 2359vw, 1568vs, 1471m, 1271m, 1049s, 820m, 586m, 427w. Anal. calc. for $C_{10}H_{16}N_4CoCl_2$: C, 37.29; H, 5.01; N, 17.39 %. Found C, 37.20; H, 4.99; N, 17.34 %. UV/Vis (CH_3CN): λ_{max}/nm ($\varepsilon/(L \cdot mol^{-1} \cdot cm^{-1})$) = 211(13 655), 580(367), 617(576)

dichloro[bis(3,5-dimethylpirazol-NN)]copper(II) (**2**)

This complex was prepared by using a modified procedure [2]. A solution of 3,5-dimethylpyrazol (2.08 mmol; 199.6 mg) in 3 mL of acetone was added to a solution of $CuCl_2$ (1.03 mmol; 138.9 mg) in 10 mL of acetone. The reaction mixture was stirred at r.t for 1 h. The green solid formed was filtered off, washed with acetone and dried under vacuum. Yield: 148.3 mg (44 %). M.p: 197-198°C. IR (KBr) ν/cm^{-1}: 3265vs, 3199vs, 2921s, 2360w, 1570vs, 1412s, 1275s, 1171s, 1043s, 795s, 819s, 795s, 686s, 431m. Anal. calc. for $C_{10}H_{16}N_4Cu\ Cl_2$: C, 36.76; H, 4.94; N, 17.15 %. Found C, 36.71; H, 4.91; N, 17.19 %. UV/Vis (CH_3CN): λ_{max}/nm ($\varepsilon/(L \cdot mol^{-1} \cdot cm^{-1})$) = 218 (9 040), 293 (2 452), 401 (740).

dichloro[bis(3,5-dimethyl-1-pyrazolyl)methane-NN]cobalt(II) (**3**)

This complex was prepared using a procedure from the literature [3]. A solution of *bis(3,5-dimethyl-1-pyrazolyl)methane* (0.5 mmol; 101 mg) in 3 mL of methanol (MeOH) was added to a solution of $CoCl_2$ (0.53 mmol; 126 mg) in 2 mL of MeOH. The reaction mixture was stirred at r.t for 1 h. The blue solid formed was filtered off, washed with cool MeOH and diethyl ether and dried at 80°C for 5 h. Yield: 58.2 mg (57 %). M.p: 308-309 °C. IR (KBr) ν/cm^{-1}: 3133m, 1556s, 1465s, 1390s, 1278vs, 1051m, 807s, 677s, 493w. Anal. calc. for $C_{11}H_{16}N_4CoCl_2$: C, 39.54; H, 4.83; N, 16.77 %. Found C, 39.52; H, 4.76; N, 16.74 %. UV/Vis (CH_3CN): λ_{max}/nm (ε/(L · mol^{-1} · cm^{-1})) = 212 (13 649), 257 (4 509), 565 (338), 612 (362), 668 (424).

dichloro[bis(3,5-dimethyl-1-pyrazolyl)methane-NN]copper(II) (**4**)

This complex was prepared by using a modified procedure from the literature [4]. A solution of *bis(3,5-dimethyl-1-pyrazolyl)methane* (1.47 mmol; 300 mg) in 6 mL of acetone was added to a solution of $CuCl_2$ (1.48 mmol; 253 mg) in 2 mL of acetone. The reaction mixture was stirred at r.t for 30 min. The yellow solid formed was filtered off, washed with acetone and diethyl ether and dried at 80°C for 6 h. Yield: 243 mg (78%). M.p: 206-207 °C. IR (KBr) ν/cm^{-1}: 3027m, 1558s, 1467s, 1386s, 1279vs, 1044m, 785s, 677s, 492w. Anal. calc. for $C_{11}H_{16}N_4CuCl_2$: C, 39.01; H, 4.76; N, 16.54 %. Found C, 39.01; H, 4.75; N, 16.54 %. UV/Vis (CH_3CN): λ_{max}/nm (ε/(L · mol^{-1} · cm^{-1})) = 220 (12 792), 269 (1 803), 306 (1 798), 457 (609)

dichloro[bis(1,2,4-triazol-1-yl)methane-NN]cobalt(II) (**9**)

This complex was prepared by using a modified procedure from Lobbia et al [5]. A solution of 1,2,4-triazol-1-yl)methane (0.67 mmol; 101.0 mg) in 2 mL of ethanol was added to a solution of $CoCl_2$ (0.67 mmol; 86.8 mg) in 5 mL of acetone. The reaction mixture was stirred at r.t for 10 h. The blue solid formed was filtered off, washed with ethanol and acetone and dried under vacuum. Yield: 155 mg (82.5 %). M.p: 397-308 °C. IR (KBr) ν/cm^{-1}: 3115m, 2094m, 1518s,

1405w, 1283s, 1209s, 1126vs, 983m, 888w, 737m, 676m, 418vw. Anal. calc. for $C_5H_6N_6CoCl_2$: C, 21.45; H, 2.16; N, 30.02 %. Found C, 21.40; H, 2.16; N, 30.01 %. UV/Vis (DMSO): λ_{max}/nm (ε/(L · mol^{-1} · cm^{-1})) = 613 (77), 679 (132)

dichloro[bis(1,2,4-triazol-1-yl) methane-NN]copper(II) (**10**)

This complex was prepared by modified literature procedures [5]. A solution of 1,2,4-triazol-1-yl)methane (1.33 mmol; 199.9 mg) in 3 mL of acetone was added to a solution of $CuCl_2$ (1.36 mmol; 182.5 mg) in 10 mL of acetone. The reaction mixture was stirred at r.t for 30 min. The light blue solid formed was filtered off, washed with acetone and dried at 80 °C for 12 h. Yield: 344 mg (91.0 %). M.p: 267-269 °C. IR (KBr) ν/cm^{-1}: 3448w, 3137m, 1525m, 1460w, 1387w, 1287m, 1212m, 1135s, 1114vs, 1032m, 961m, 780m, 739s, 668s, 633w. Anal. calc. for $C_5H_6N_6CuCl_2$: C, 21.10; H, 2.13; N, 29.53 %. Found C, 21.10; H, 2.11; N, 29.33 %. UV/Vis (DMSO): λ_{max}/nm (ε/(L · mol^{-1} · cm^{-1})) = 291 (3 263)

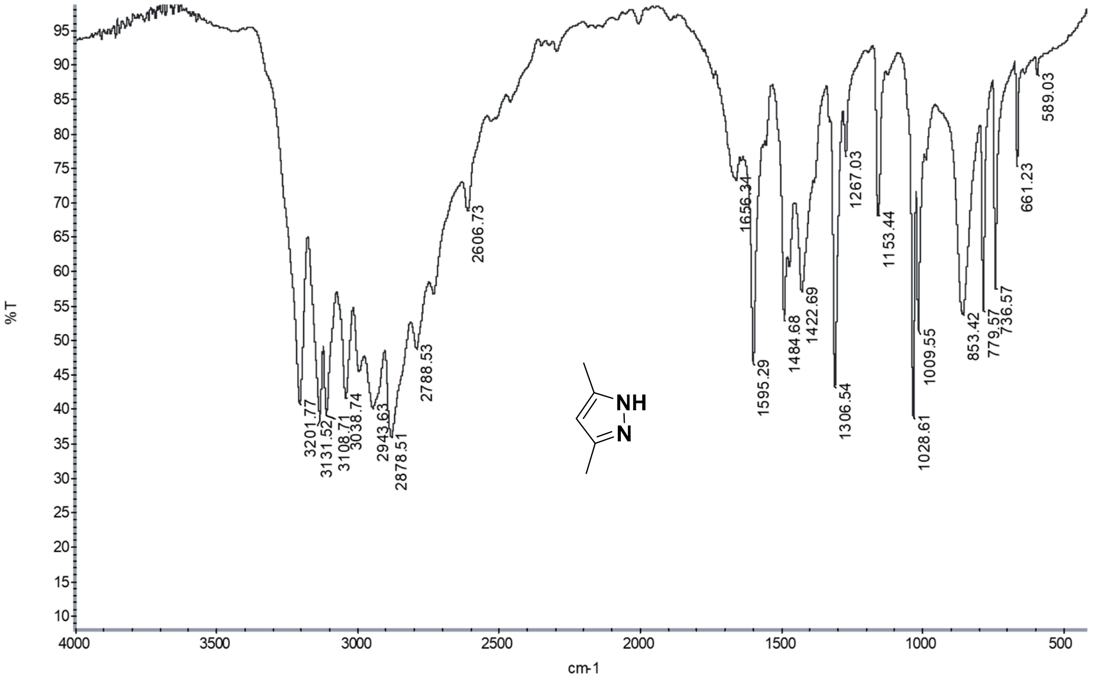

Figure S1. IR spectrum of 3,5-dimethylpyrazole

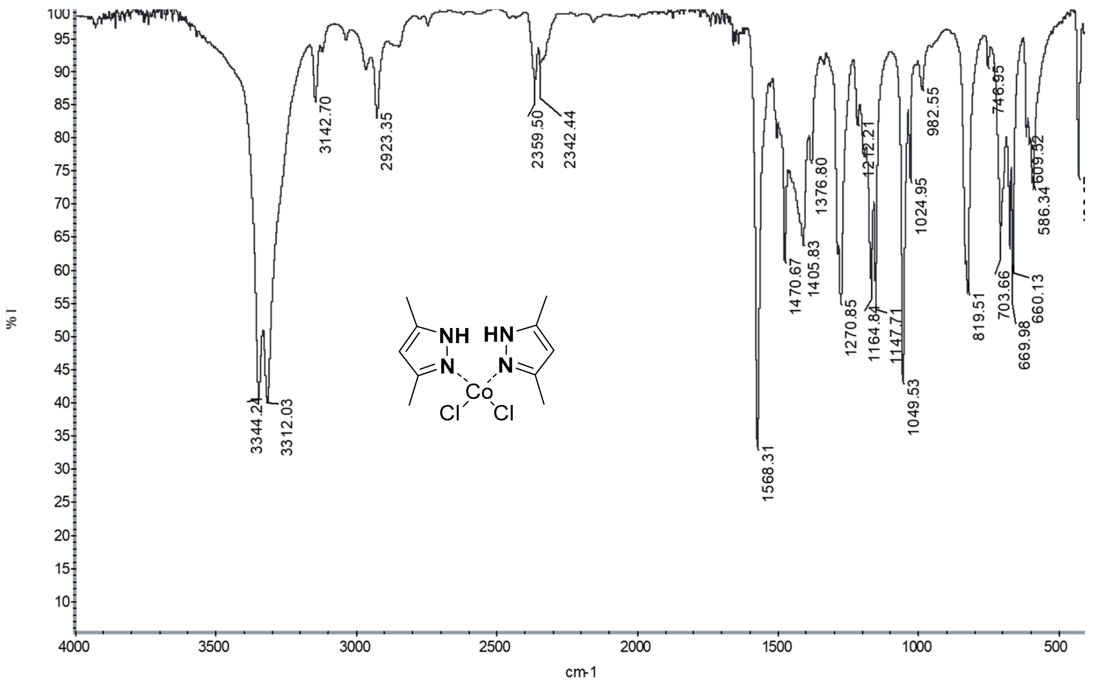

Figure S2. IR spectrum of dichloro[bis(3,5-dimethylpirazol-NN)]cobalt(II) (1)

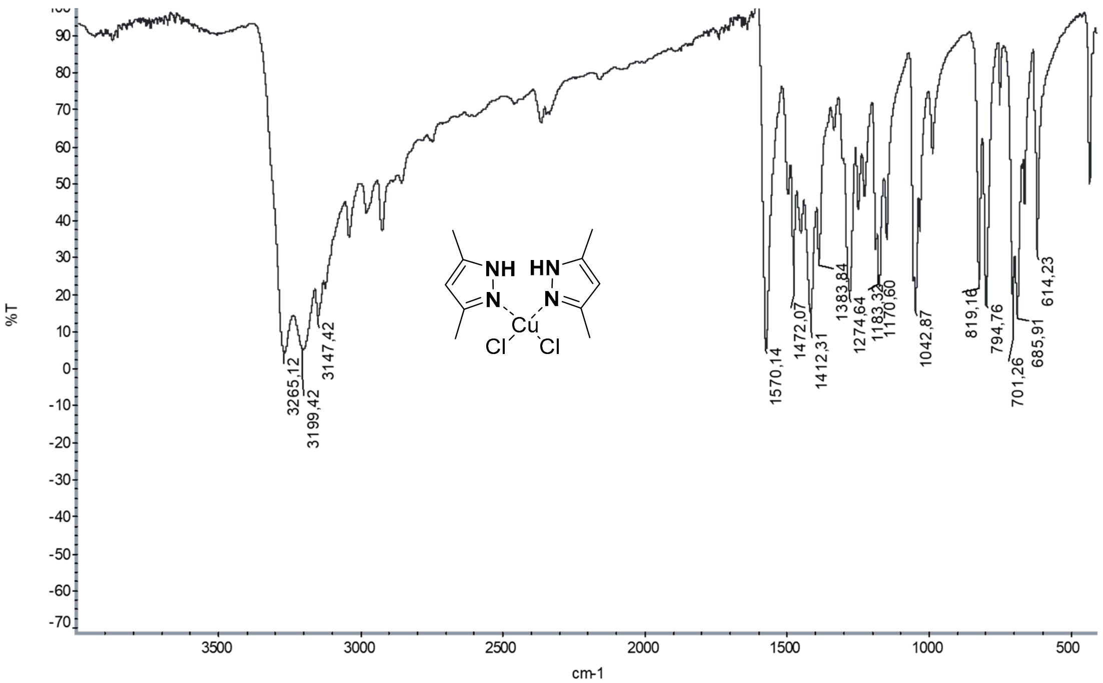

Figure S3. IR spectrum of dichloro[bis(3,5-dimethylpirazol-NN)]copper(II) (2)

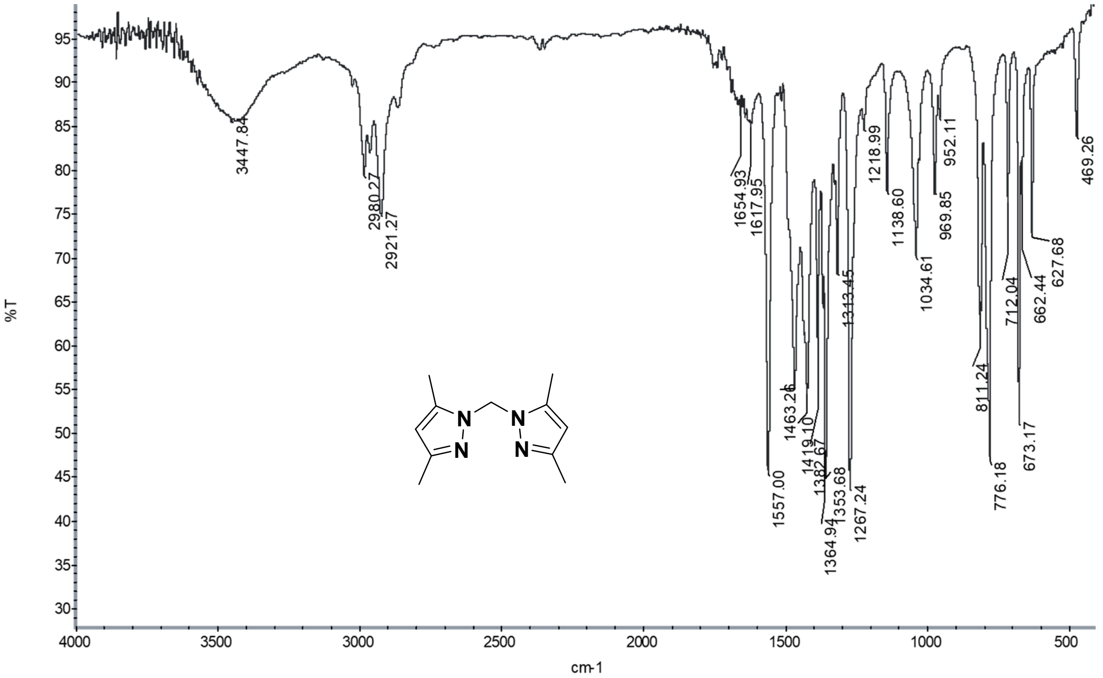

Figure S4. IR spectrum of bis(3,5-dimethyl-1-pyrazolyl)methane

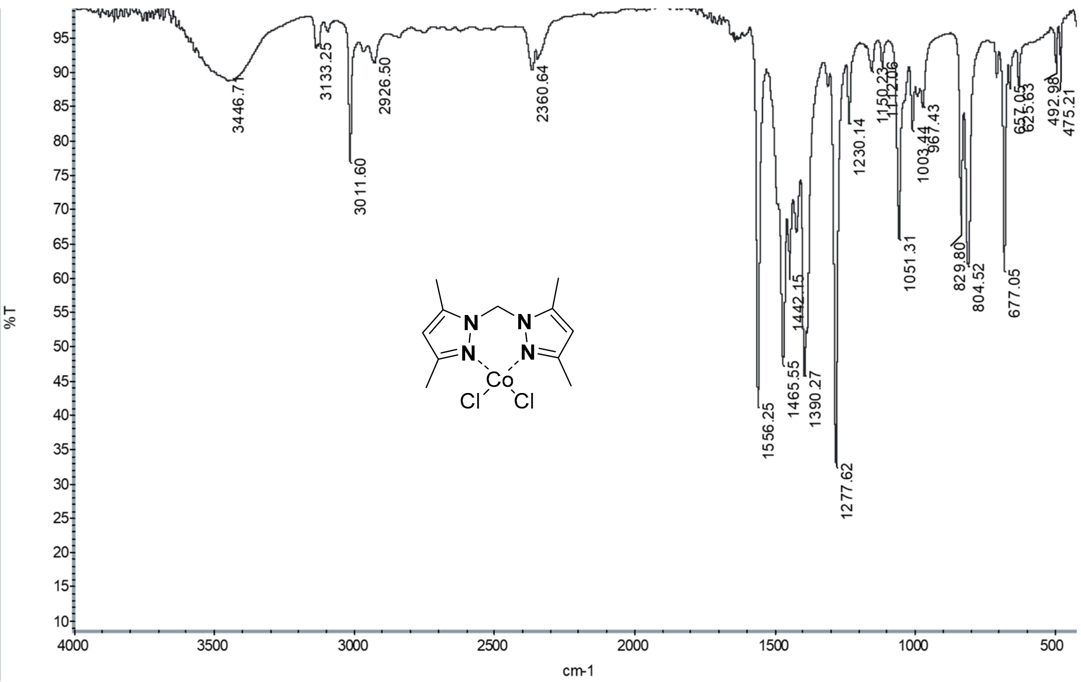

Figure S5. IR spectrum of dichloro[bis(3,5-dimethyl-1-pyrazolyl)methane-NN]cobalt(II) (3)

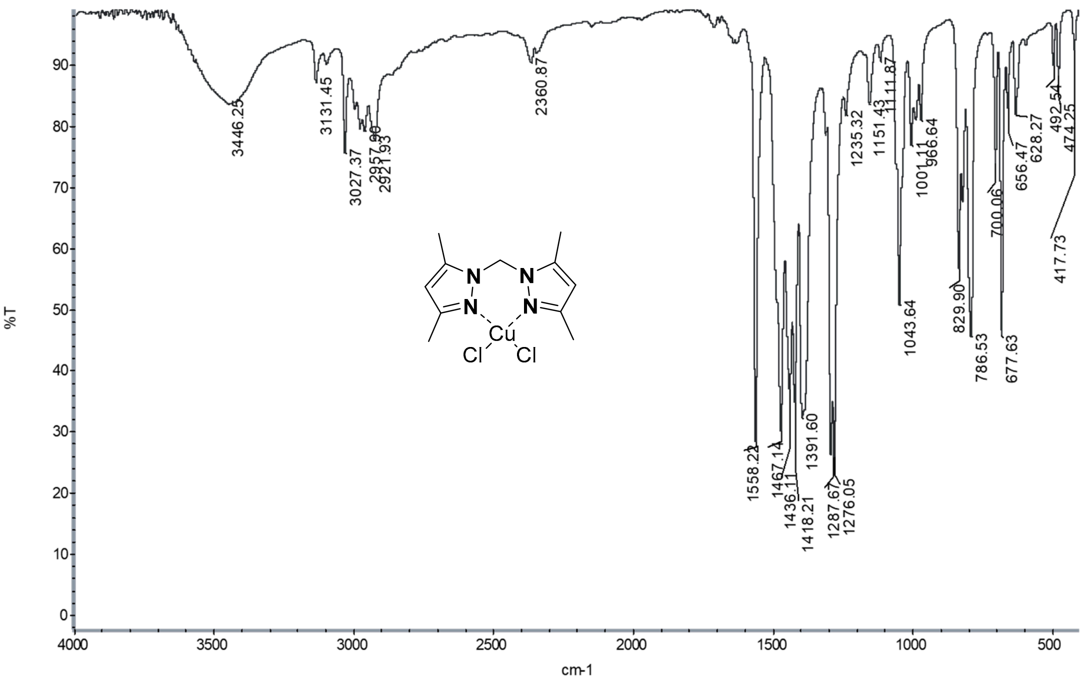

Figure S6. IR spectrum of dichloro[bis(3,5-dimethyl-1-pyrazolyl)methane-NN]copper(II) (4)

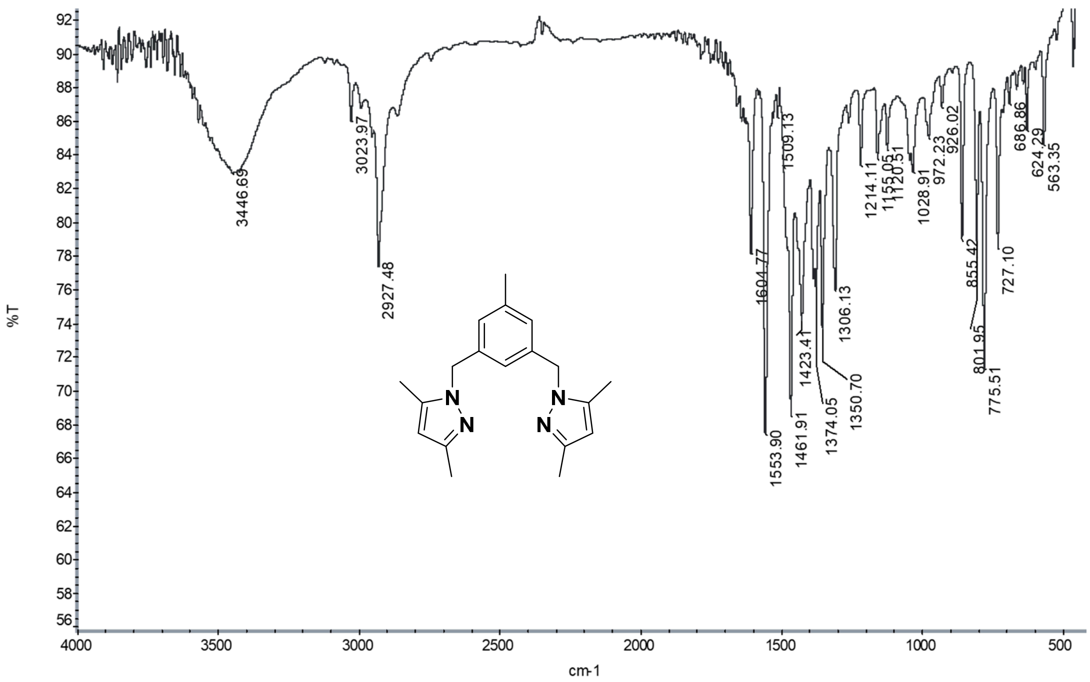

Figure S7. IR spectrum of 3,5-bis(3,5-dimethylpyrazol-1-ylmethyl)toluene

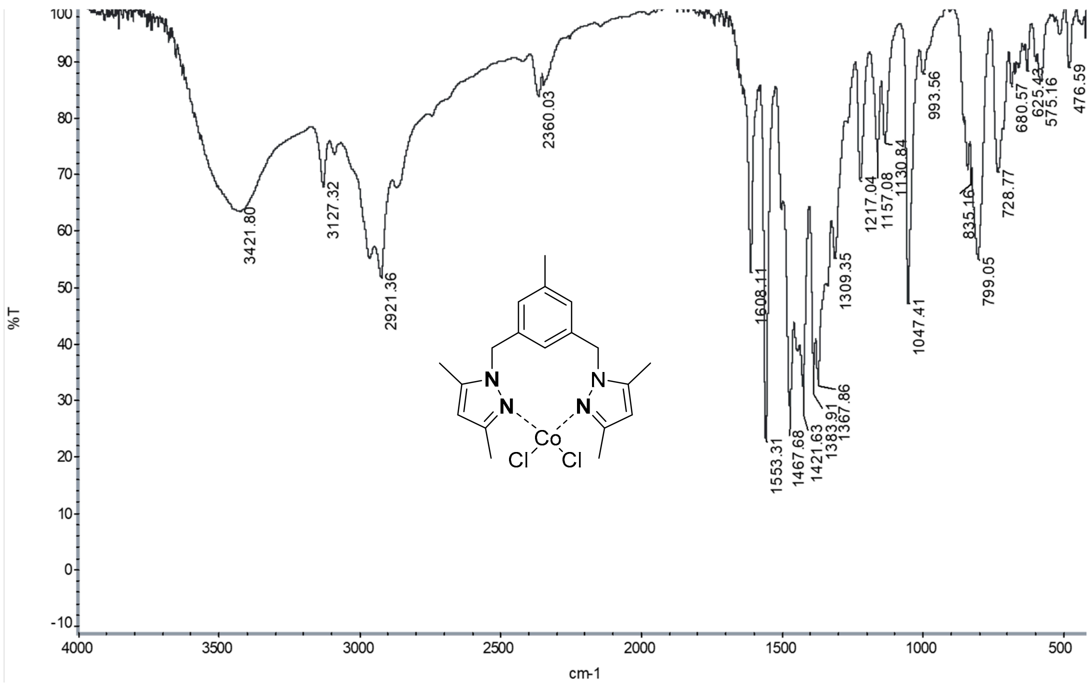

Figure S8. IR spectrum of dichloro[3,5-bis(3,5-dimethylpyrazol-1-ylmethyl)toluene-NN]cobalt(II) (5)

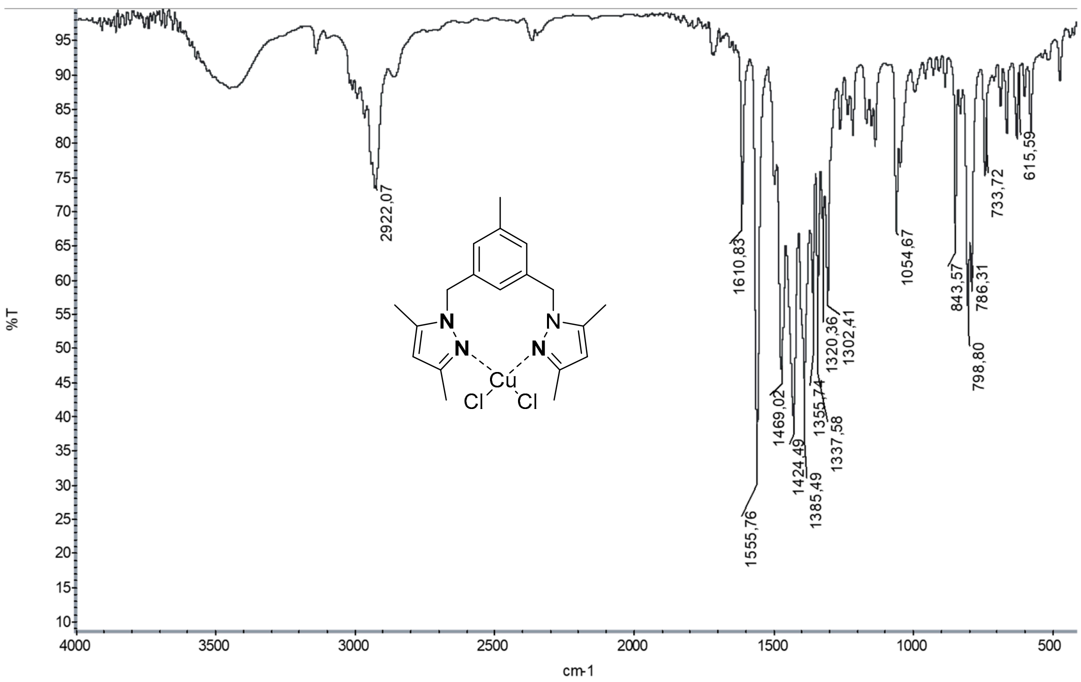

Figure S9. IR spectrum of dichloro[3,5-bis(3,5-dimethylpyrazol-1-ylmethyl)toluene-NN]copper(II) (6)

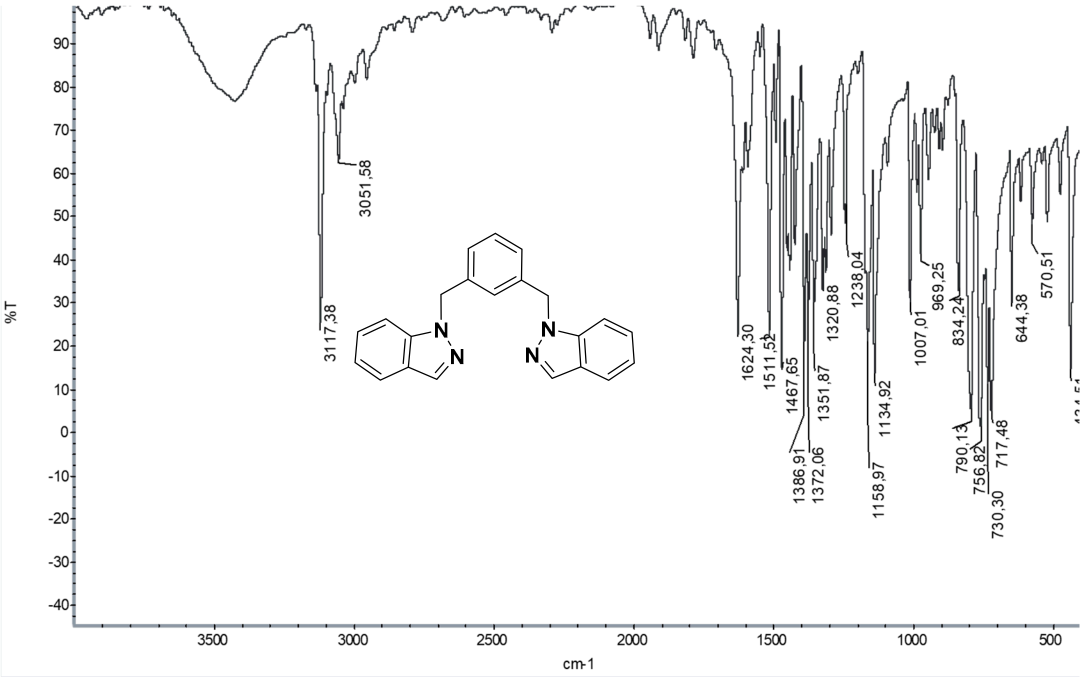

Figure S10. IR spectrum of 1,3-bis(indazol-1-ylmethyl)benzene

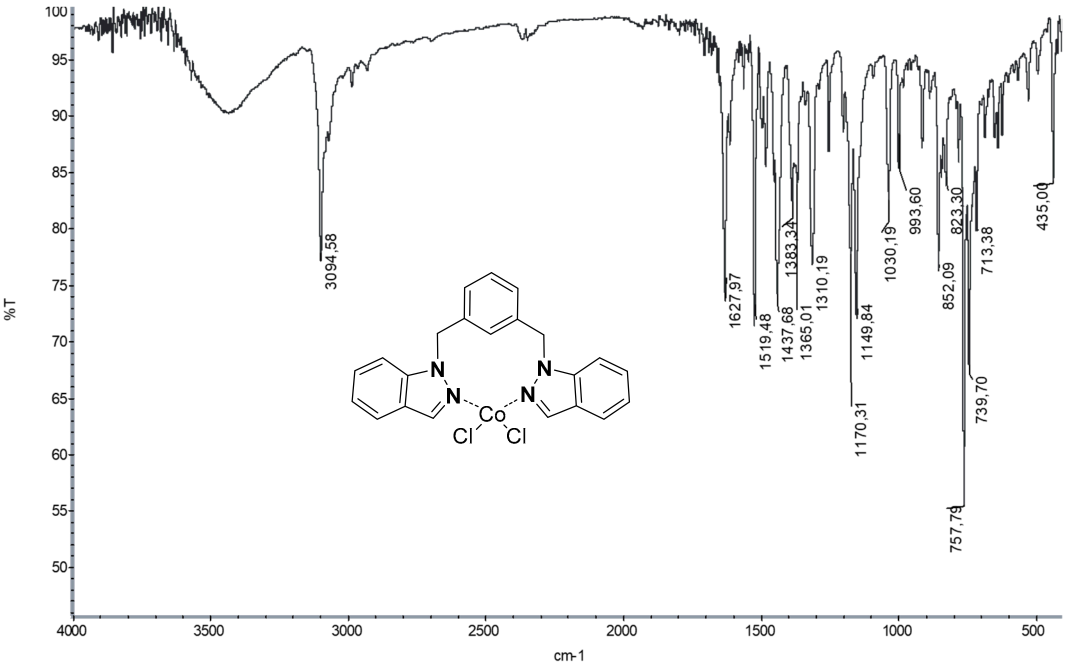

Figure S11. IR spectrum of dichloro[1,3-bis(indazol-1-ylmethyl)benzene-NN]cobalt(II) (7)

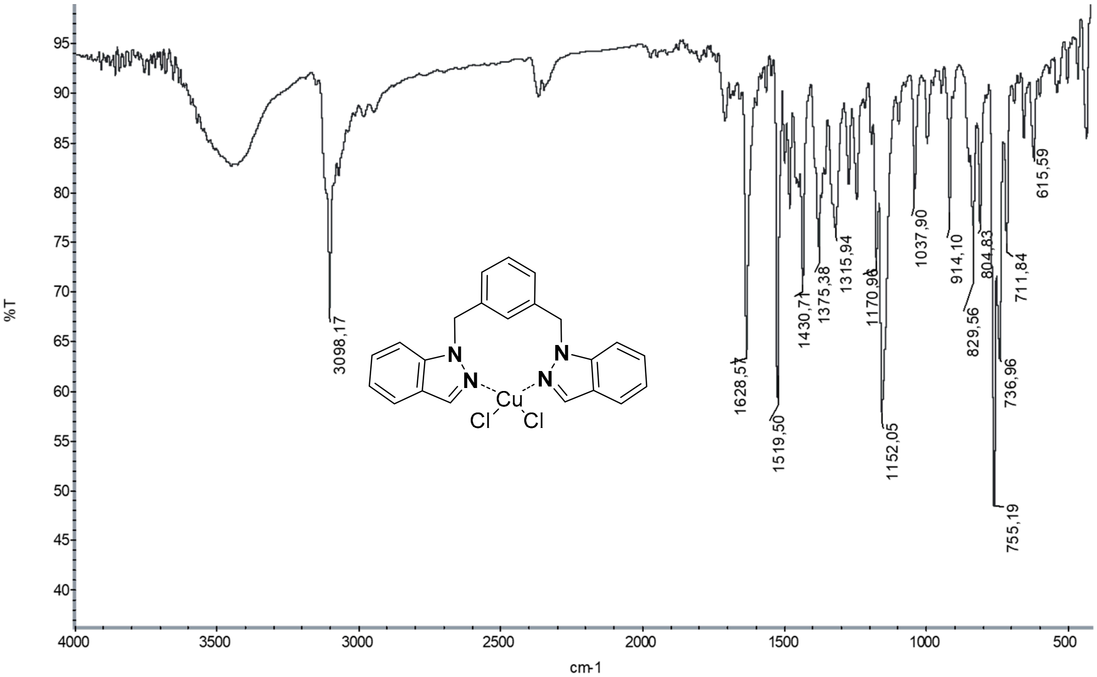

Figure S12. IR spectrum of dichloro[1,3-bis(indazol-1-ylmethyl)benzene-NN]copper(II) (8)

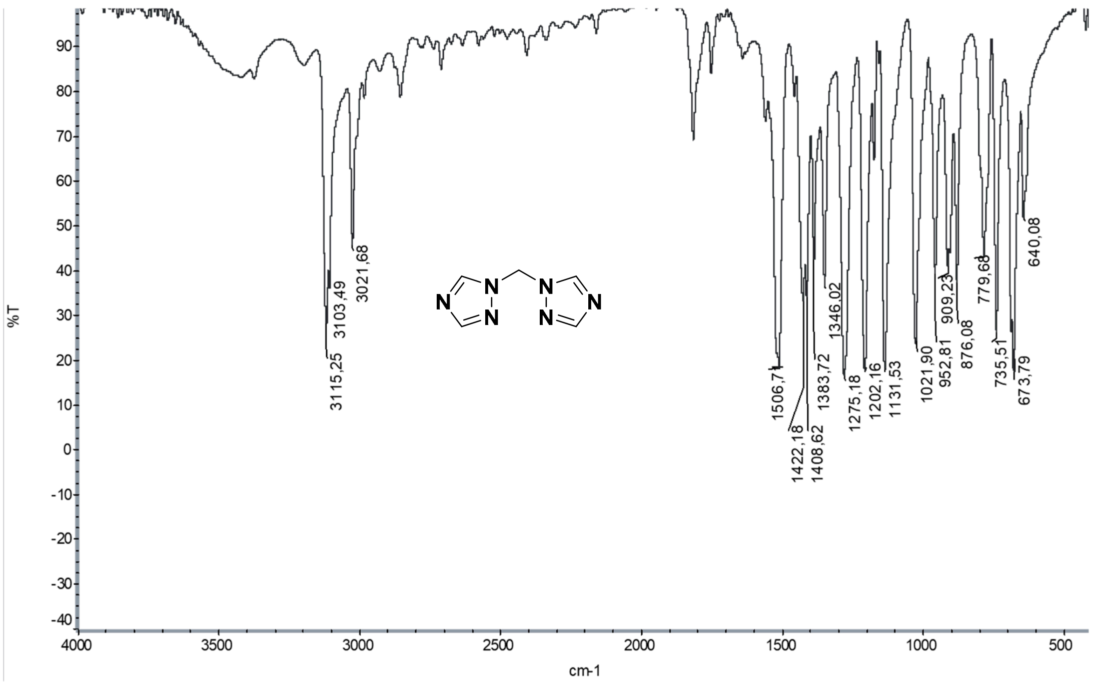

Figure S13. IR spectrum of bis(1,2,4-triazol-1-yl)methane

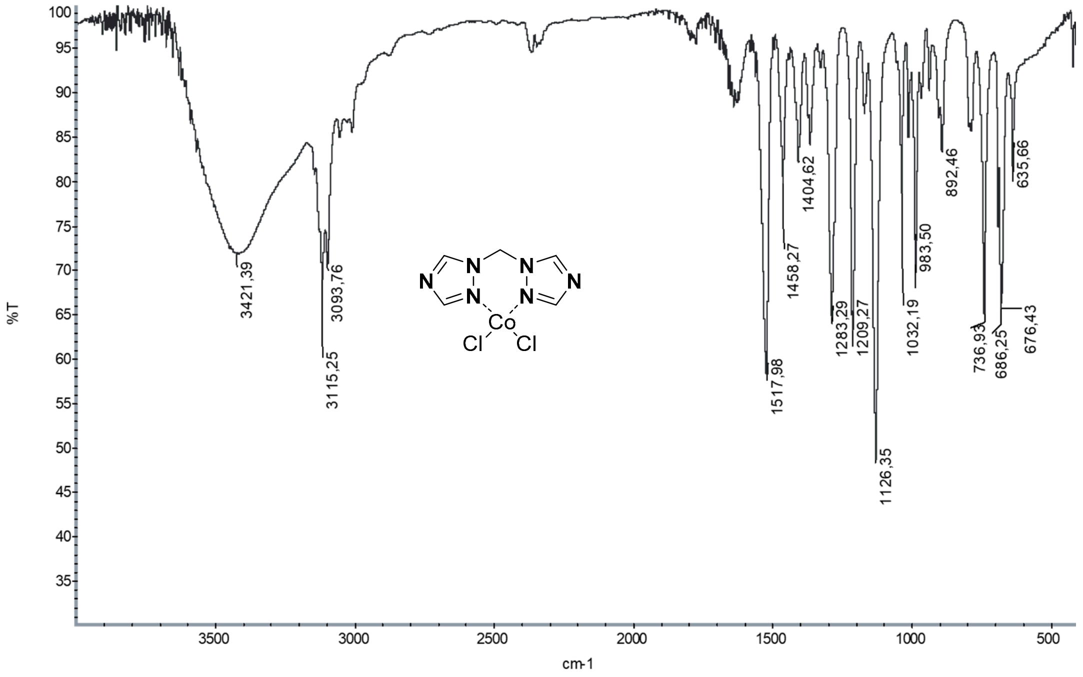

Figure S14. IR spectrum of dichloro[bis(1,2,4-triazol-1-yl)methane-NN]cobalt(II) (9)

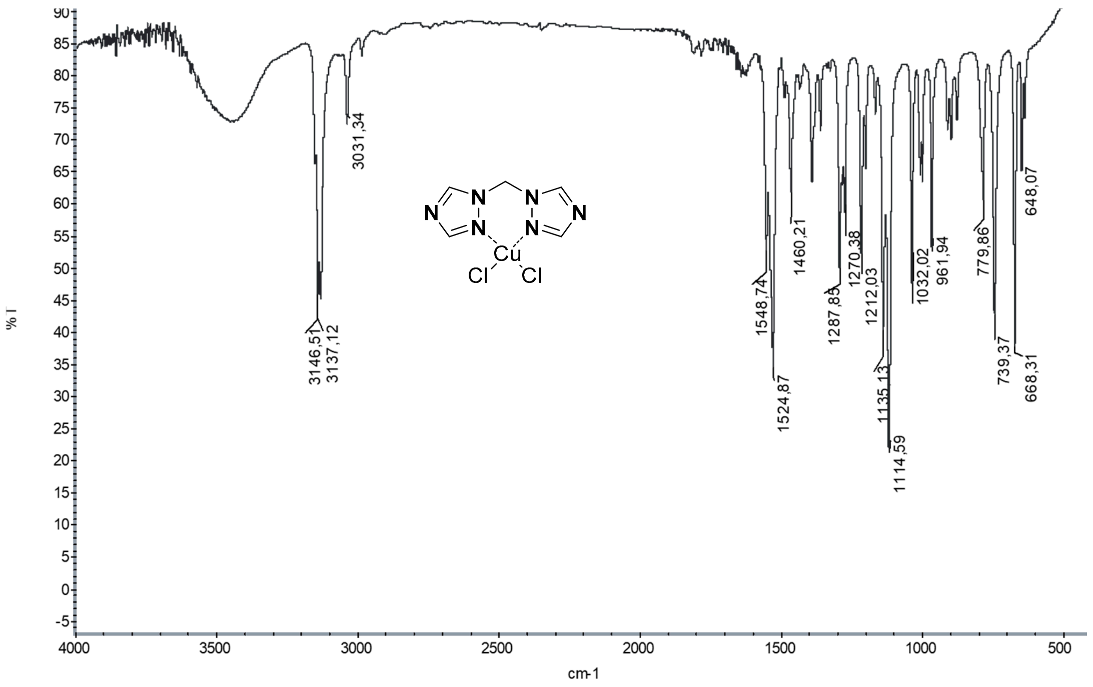

Figure S15. IR spectrum of dichloro[bis(1,2,4-triazol-1-yl) methane-NN]copper(II) (10)

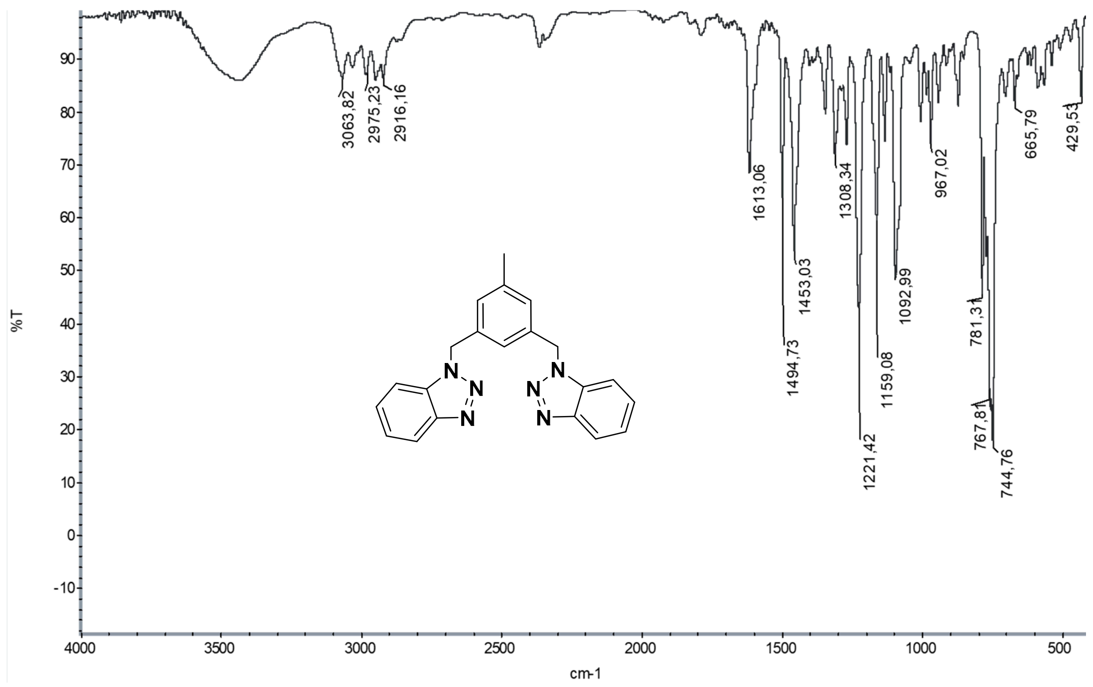

Figure S16. IR spectrum of 3,5-bis(benzotriazol-1-ylmethyl)toluene

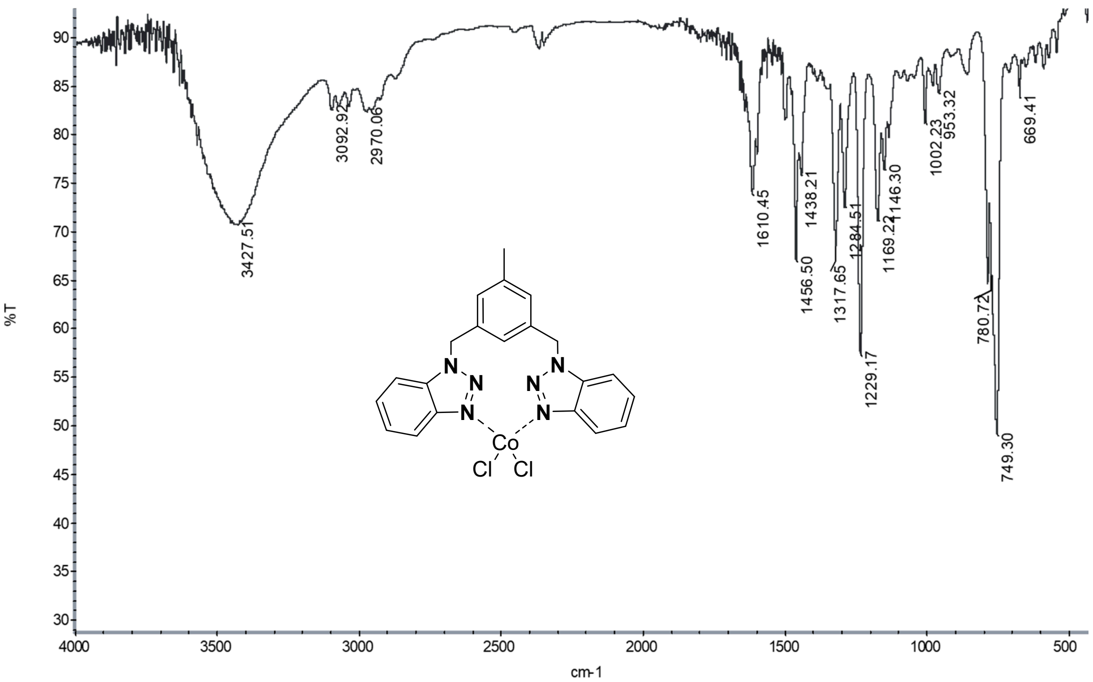

Figure S17. IR spectrum of dichloro[3,5-bis(benzotriazol-1-ylmethyl)toluene-NN]cobalt(II) (11)

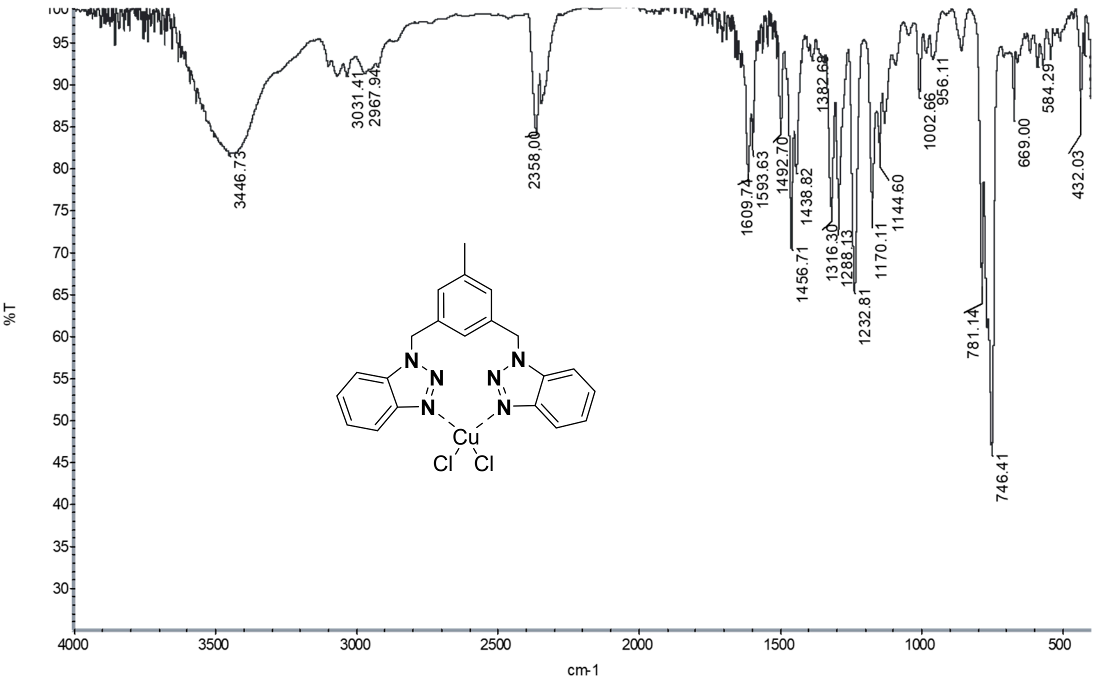

Figure S18. IR spectrum of dichloro[3,5-bis(benzotriazol-1-ylmethyl)toluene-NN]copper(II) (12)

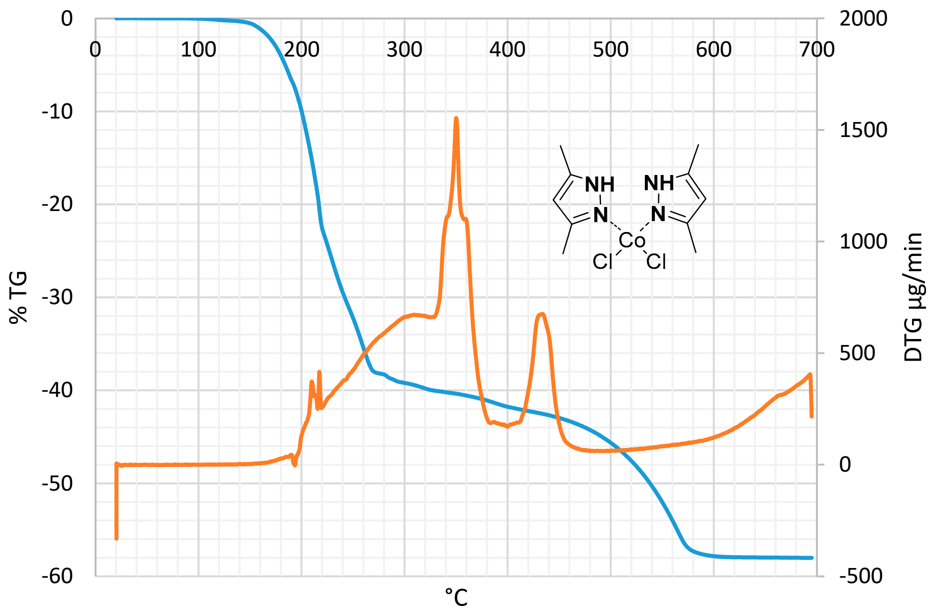

Figure S19. TGA and DTG of dichloro[bis(3,5-dimethylpirazol-NN)]cobalt(II) (1)

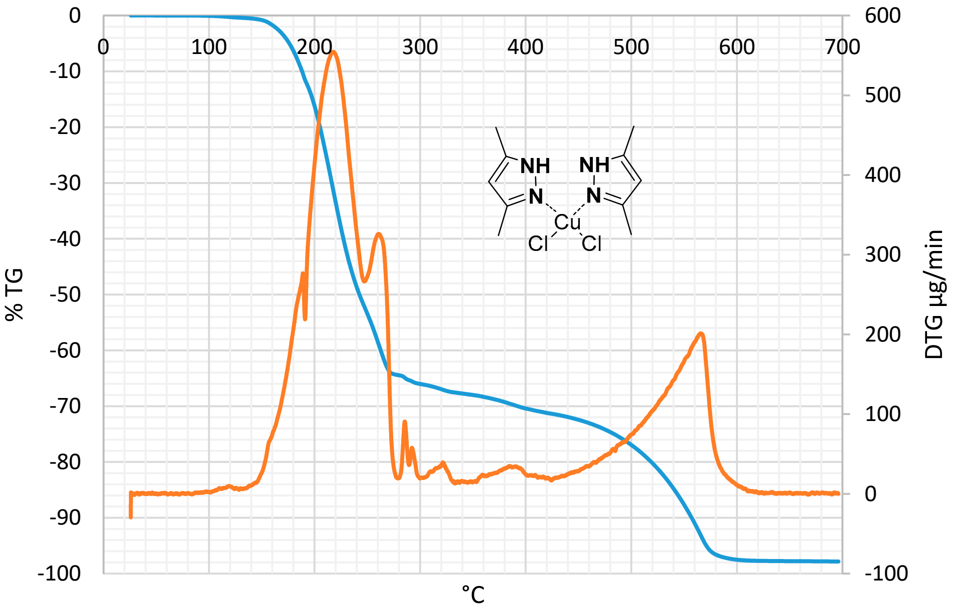

Figure S20. TGA and DTG of dichloro[bis(3,5-dimethylpirazol-NN)]copper(II) (2)

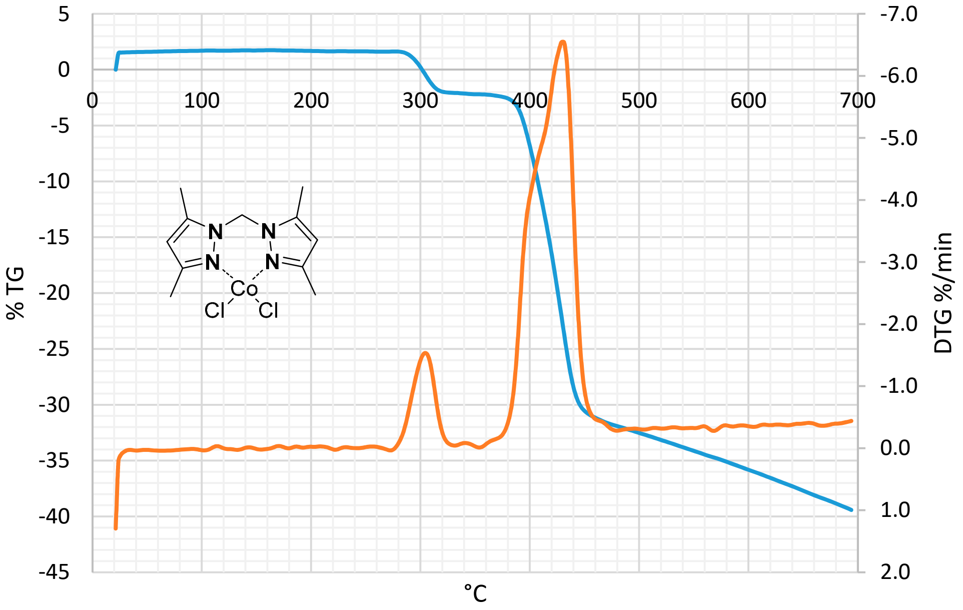

Figure S21. TGA and DTG of dichloro[bis(3,5-dimethyl-1-pyrazolyl)methane-NN]cobalt(II) (3)

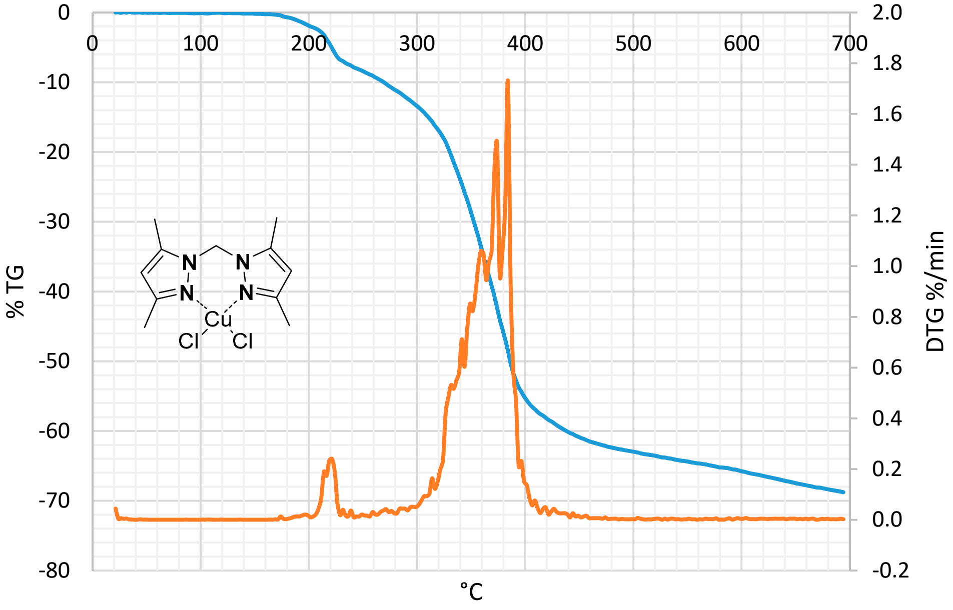

Figure S22. TGA and DTG of dichloro[bis(3,5-dimethyl-1-pyrazolyl)methane-NN]copper(II) (4)

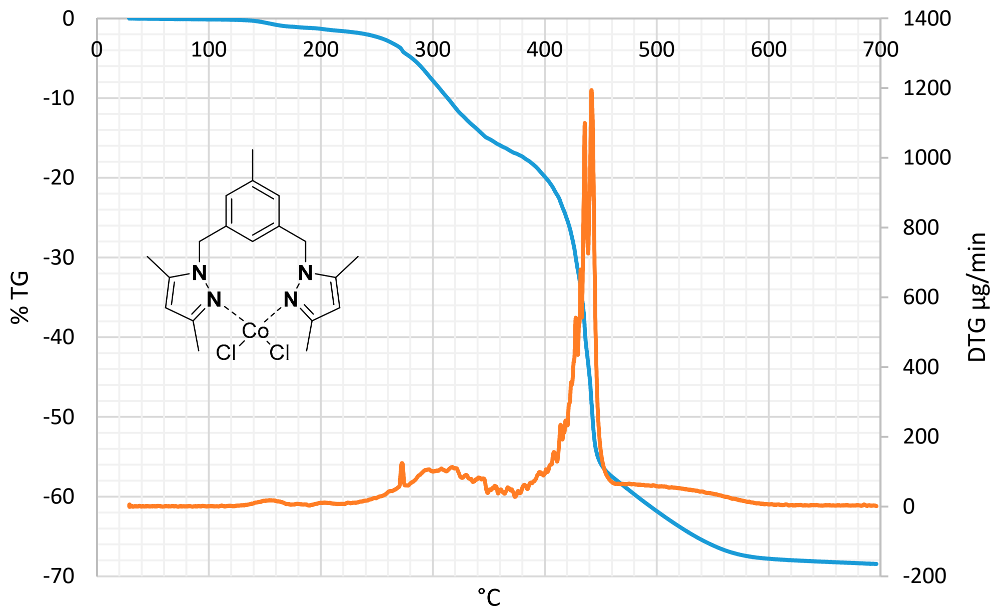

Figure S23. TGA and DTG of dichloro[3,5-bis(3,5-dimethylpyrazol-1-ylmethyl)toluene-NN]cobalt(II) (5)

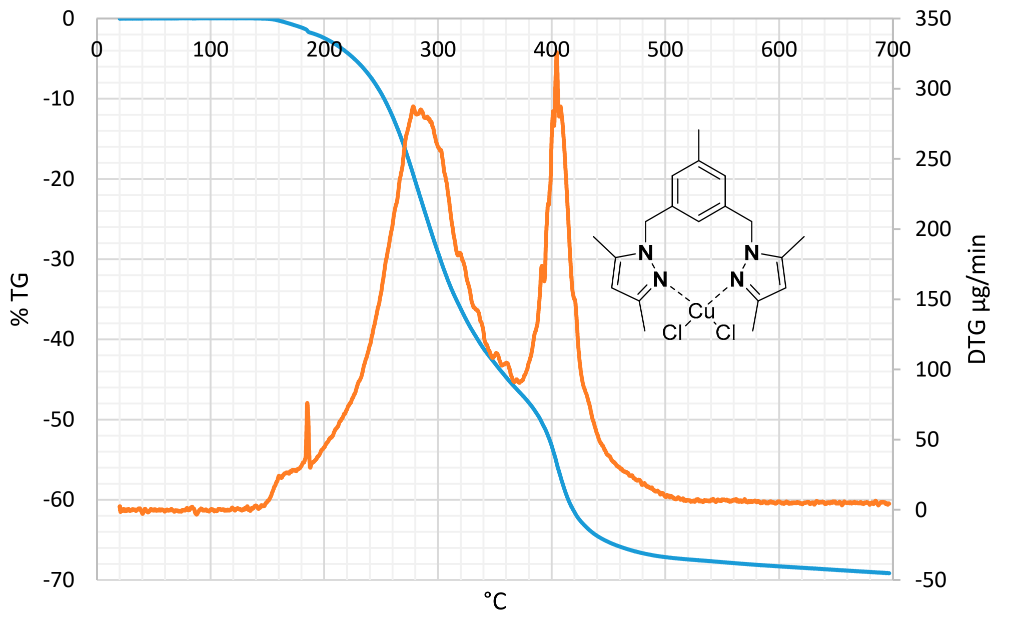

Figure 1. TGA and DTG of dichloro[3,5-bis(3,5-dimethylpyrazol-1-ylmethyl)toluene-NN]copper(II) (6)

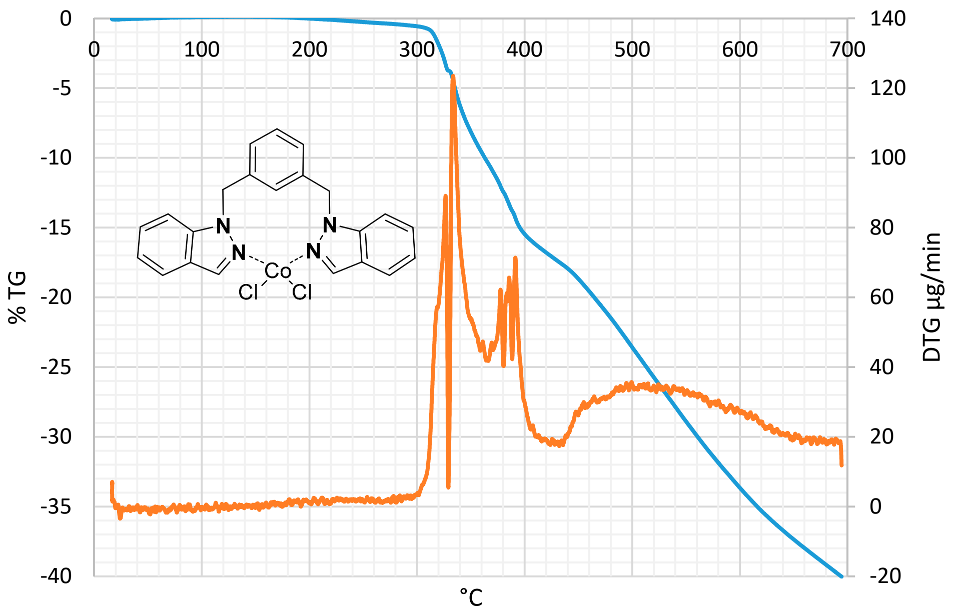

Figure S25. TGA and DTG of dichloro[1,3-bis(indazol-1-ylmethyl)benzene-NN]cobalt(II) (7)

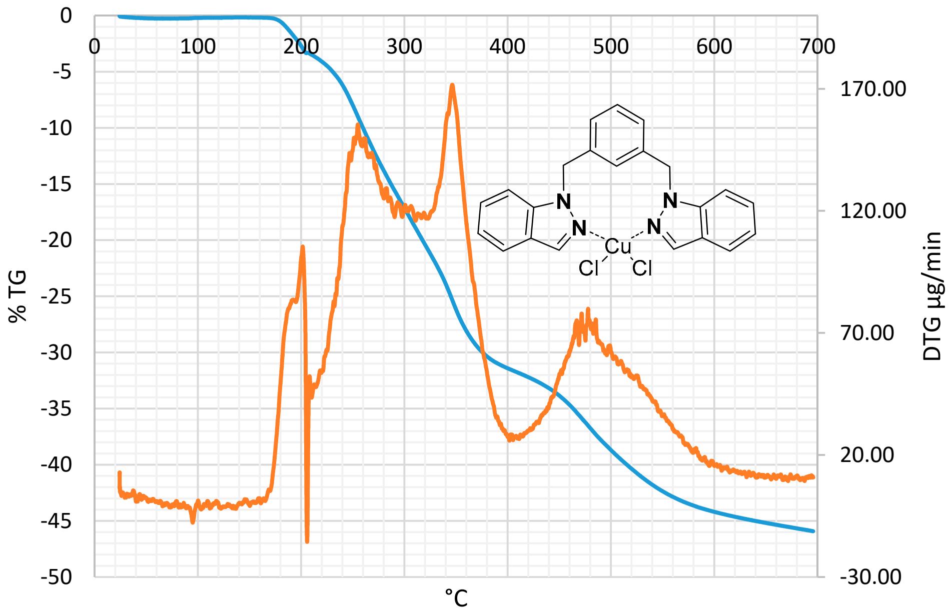

Figure S26. TGA and DTG of dichloro[1,3-bis(indazol-1-ylmethyl)benzene-NN]copper(II) (8)

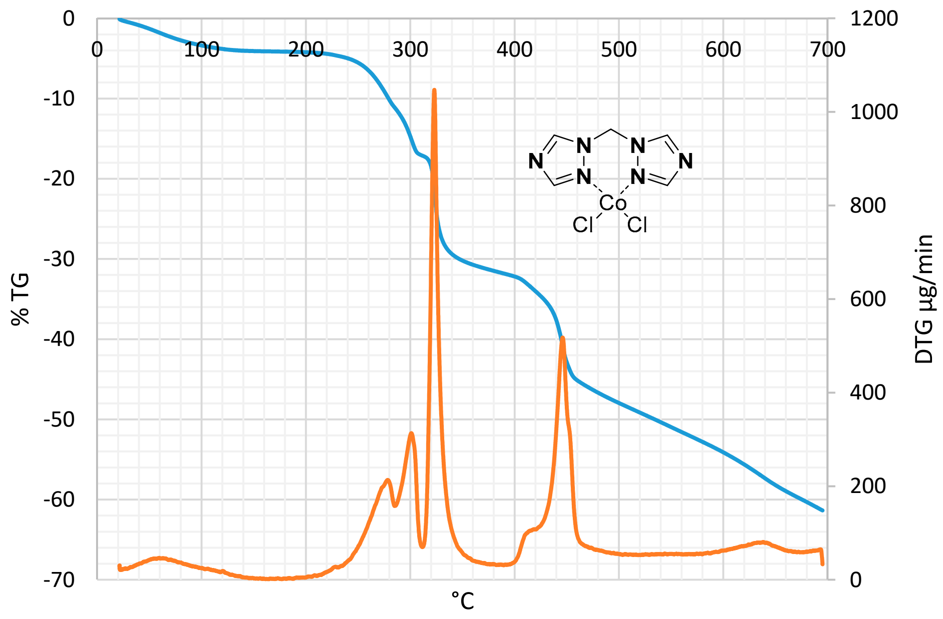

Figure S27. TGA and DTG of dichloro[bis(1,2,4-triazol-1-yl)methane-NN]cobalt(II) (9)

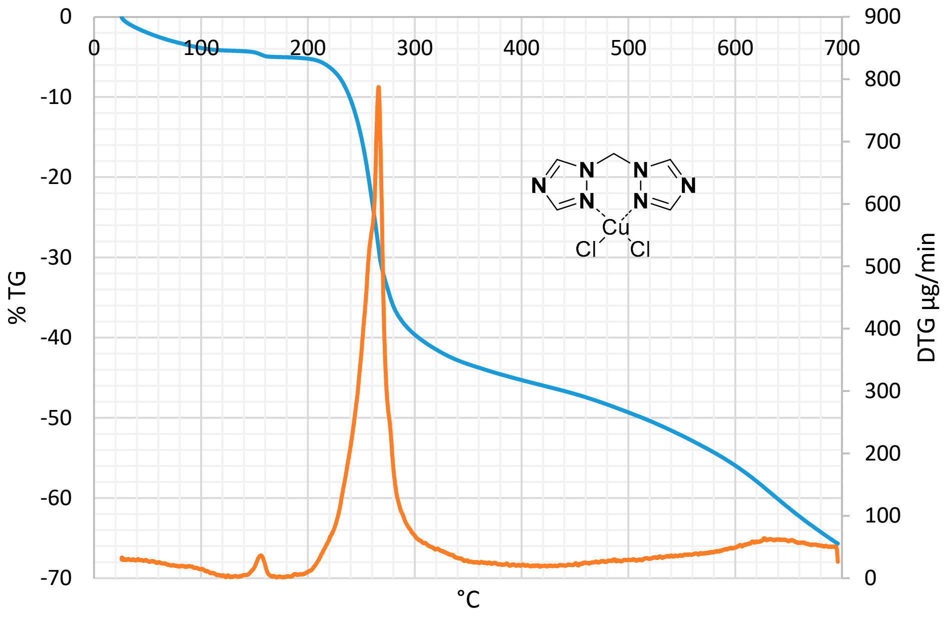

Figure S28. TGA and DTG of dichloro[bis(1,2,4-triazol-1-yl) methane-NN]copper(II) (10)

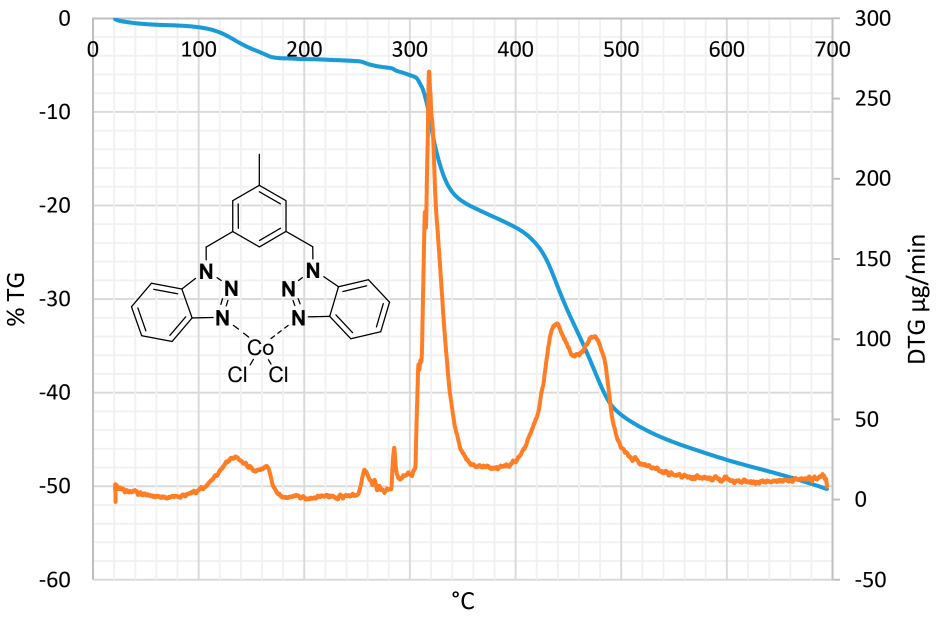

Figure S29. TGA and DTG of dichloro[3,5-bis(benzotriazol-1-ylmethyl)toluene-NN]cobalt(II) (11)

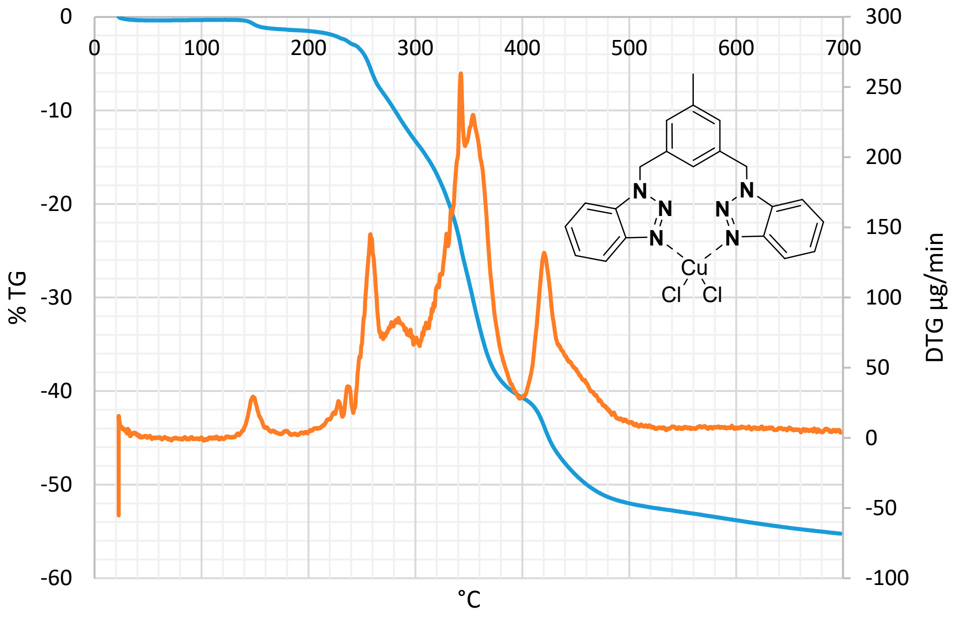

Figure S30. TGA and DTG of dichloro[3,5-bis(benzotriazol-1-ylmethyl)toluene-NN]copper(II) (12)

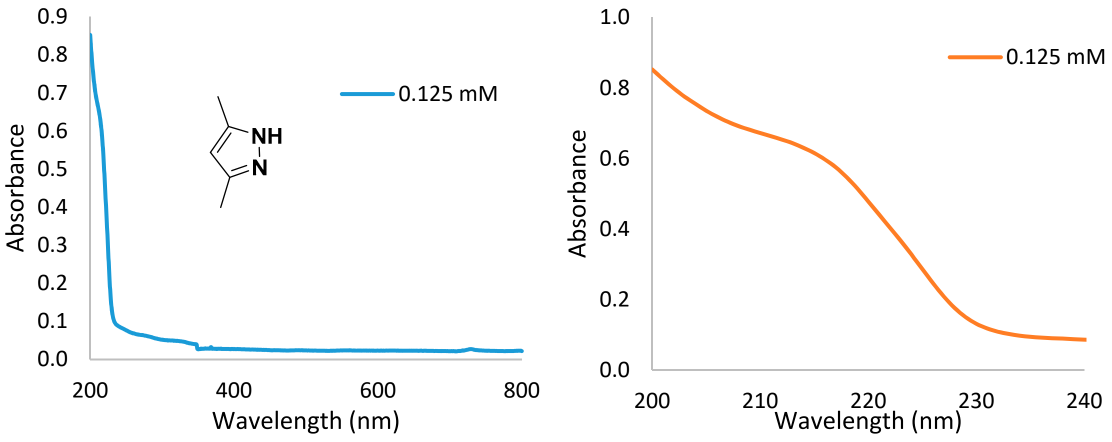

Figure S31. UV-Vis spectrum of 3,5-dimethylpyrazole in ACN

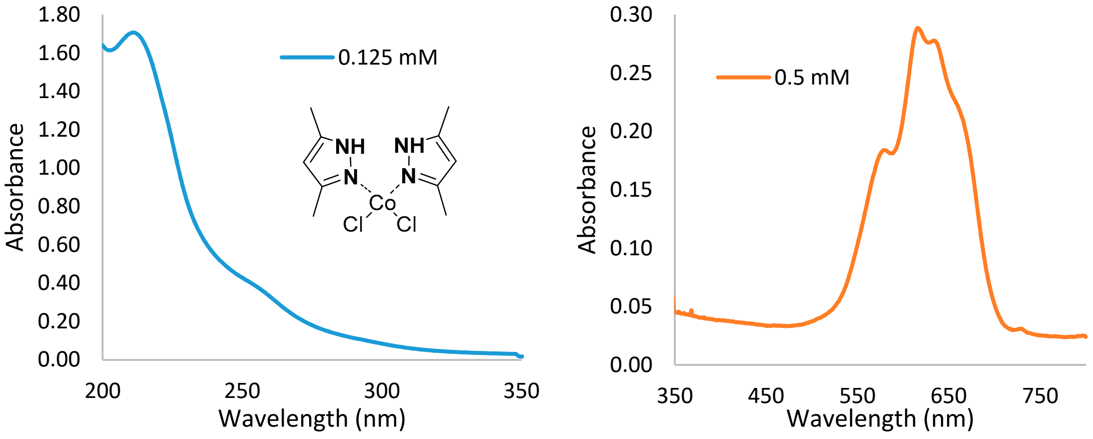

Figure S32. UV-Vis spectrum of dichloro[bis(3,5-dimethylpirazol-NN)]cobalt(II) in ACN (1)

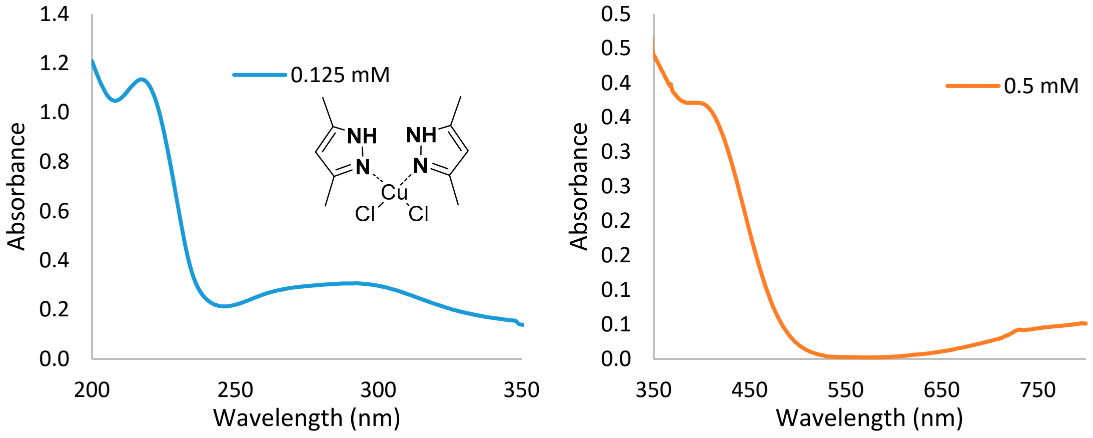

Figure S33. UV-Vis spectrum of dichloro[bis(3,5-dimethylpirazol-NN)]copper(II) in ACN (2)

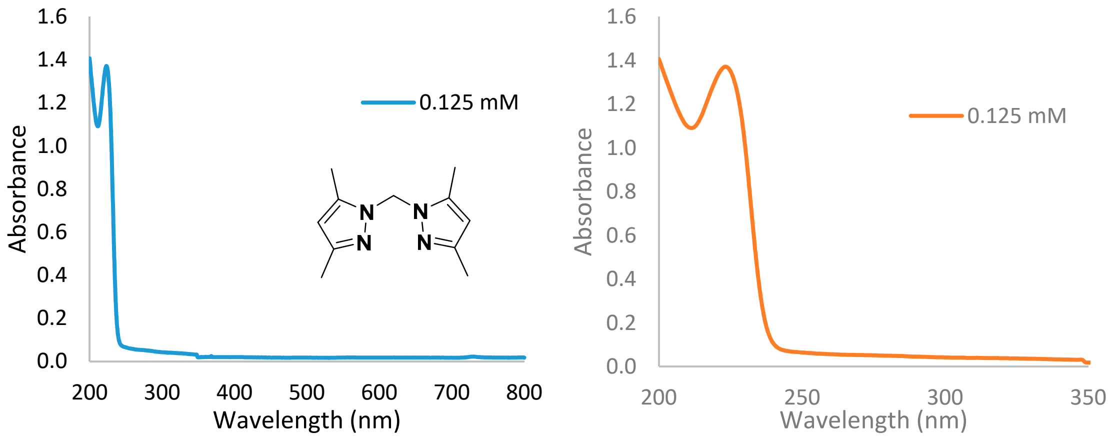

Figure S34. UV-Vis spectrum of bis(3,5-dimethyl-1-pyrazolyl)methane in ACN

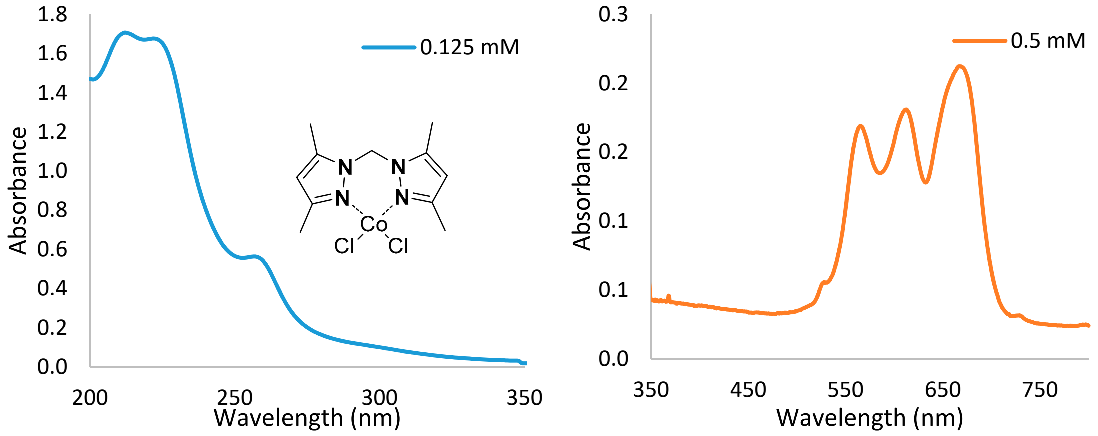

Figure S35. UV-Vis spectrum of dichloro[bis(3,5-dimethyl-1-pyrazolyl)methane-NN]cobalt(II) in ACN (3)

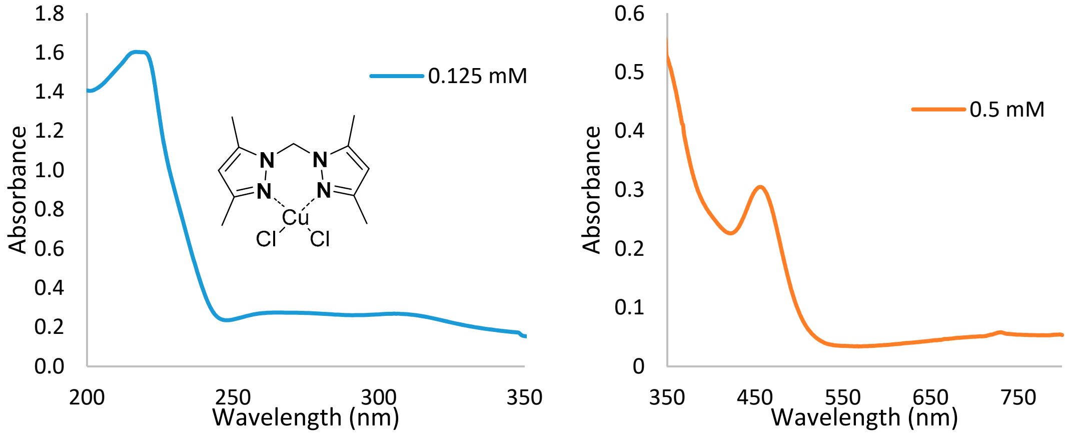

Figure S36. UV-Vis spectrum of dichloro[bis(3,5-dimethyl-1-pyrazolyl)methane-NN]copper(II) in ACN (4)

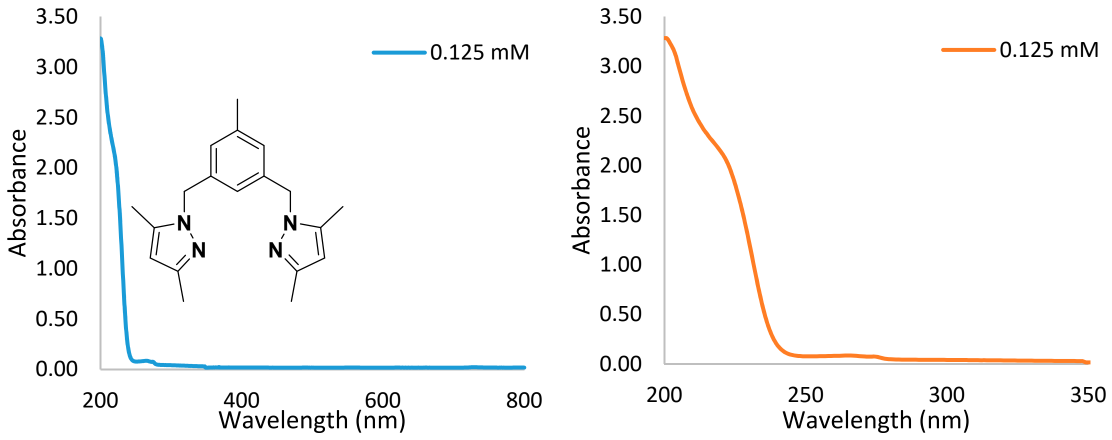

Figure S37. UV-Vis spectrum of 3,5-bis(3,5-dimethylpyrazol-1-ylmethyl)toluene in ACN

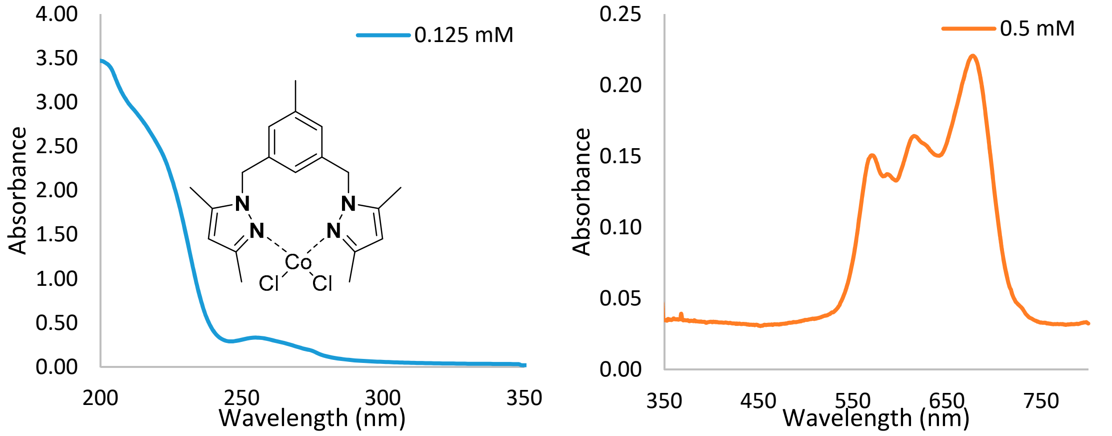

Figure S38. UV-Vis spectrum of dichloro[3,5-bis(3,5-dimethylpyrazol-1-ylmethyl)toluene-NN]cobalt(II) in ACN (5)

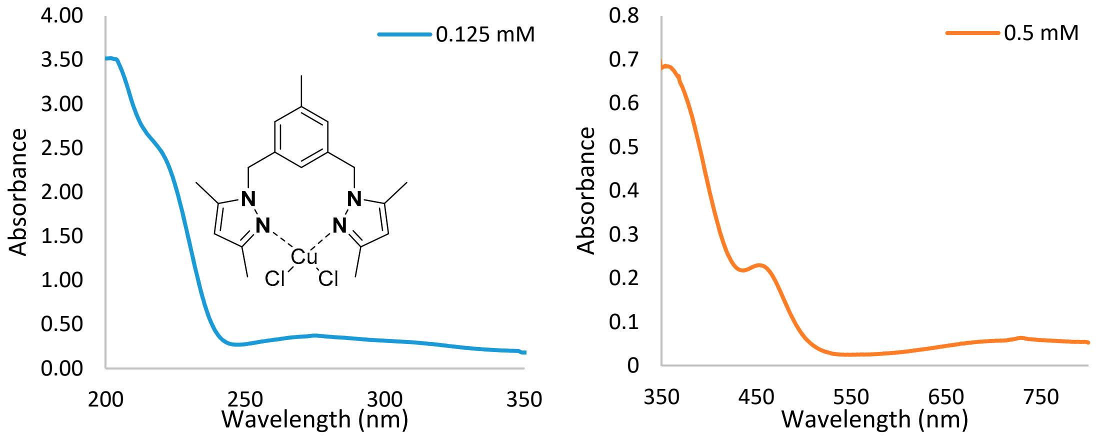

Figure S39. UV-Vis spectrum of dichloro[3,5-bis(3,5-dimethylpyrazol-1-ylmethyl)toluene-NN]copper(II) in ACN (6)

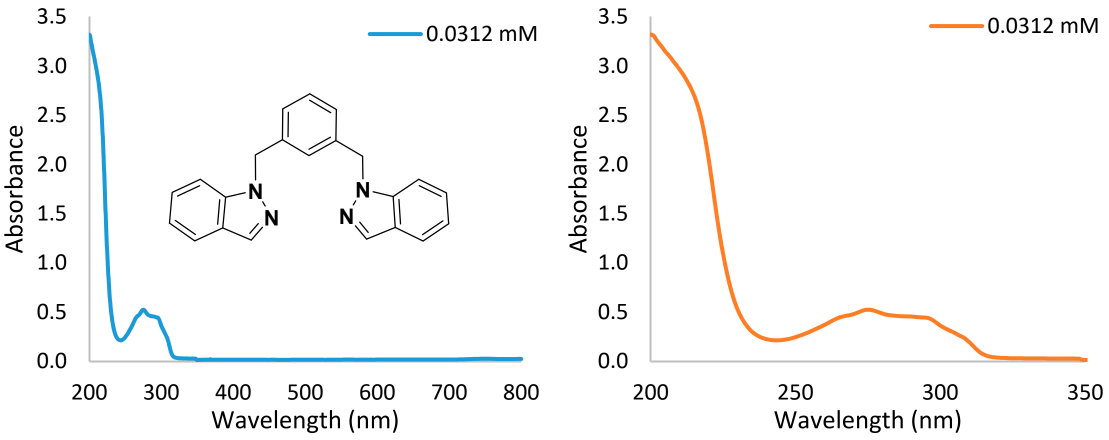

Figure S40. UV-Vis spectrum of 1,3-bis(indazol-1-ylmethyl)benzene in MeOH

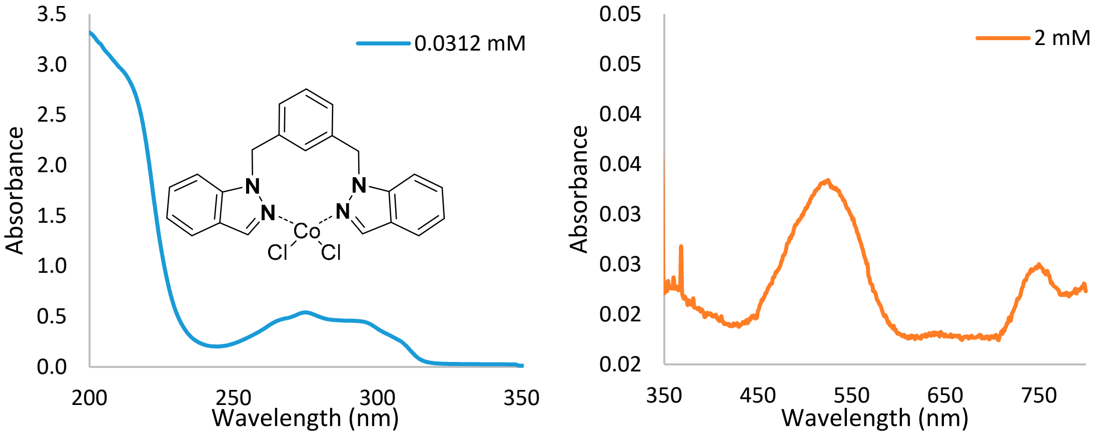

Figure S41. UV-Vis spectrum of dichloro[1,3-bis(indazol-1-ylmethyl)benzene-NN]cobalt(II) in MeOH (7)

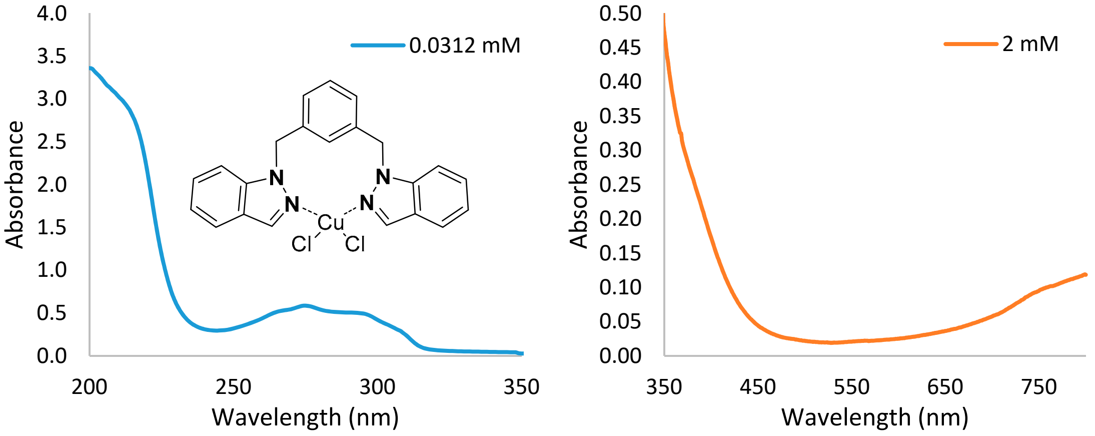

Figure S42. UV-Vis spectrum of dichloro[1,3-bis(indazol-1-ylmethyl)benzene-NN]copper(II) in MeOH (8)

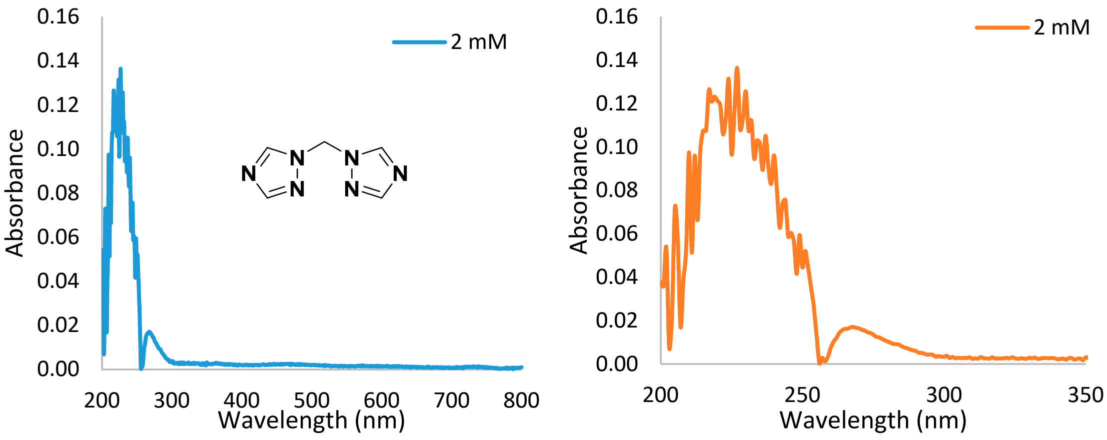

Figure S43. UV-Vis spectrum of bis(1,2,4-triazol-1-yl)methane in DMSO

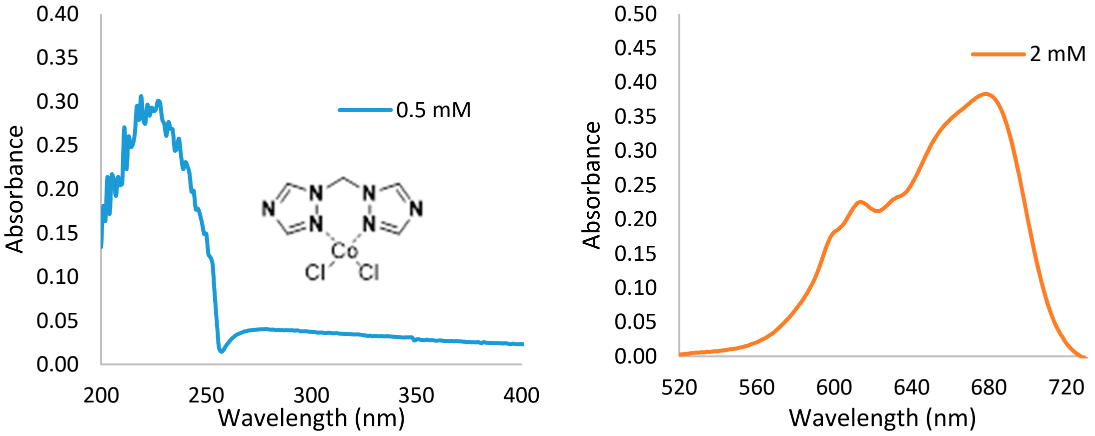

Figure S44. UV-Vis spectrum of dichloro[bis(1,2,4-triazol-1-yl)methane-NN]cobalt(II) in DMSO (9)

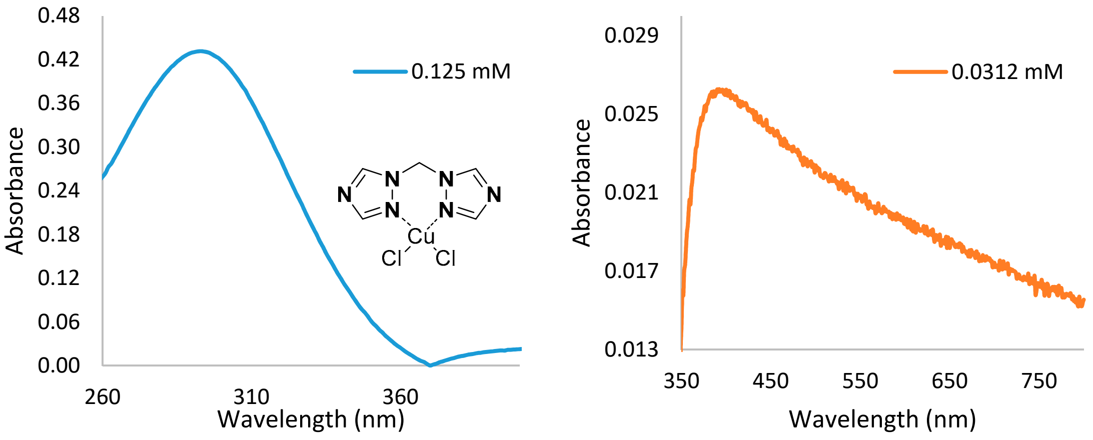

Figure S45. UV-Vis spectrum of dichloro[bis(1,2,4-triazol-1-yl) methane-NN]copper(II) in DMSO (10)

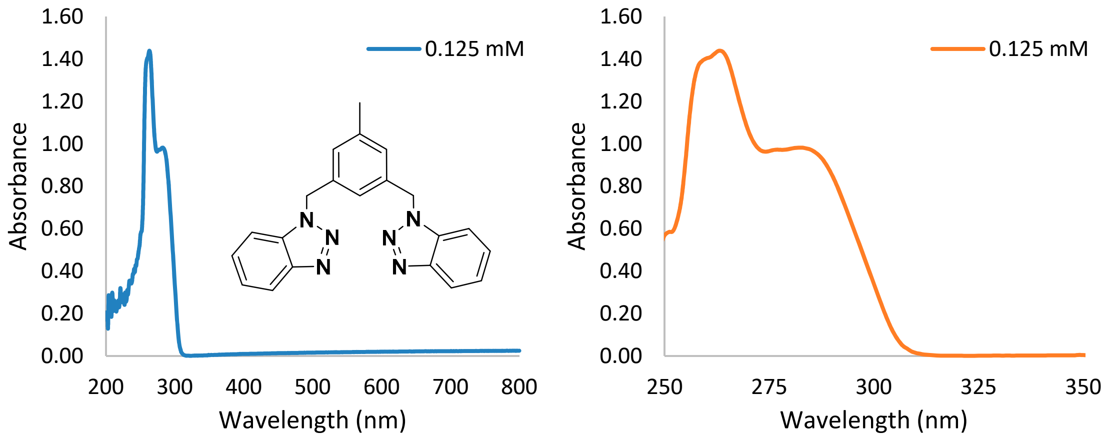

Figure S46. UV-Vis spectrum of 3,5-bis(benzotriazol-1-ylmethyl)toluene in DMSO

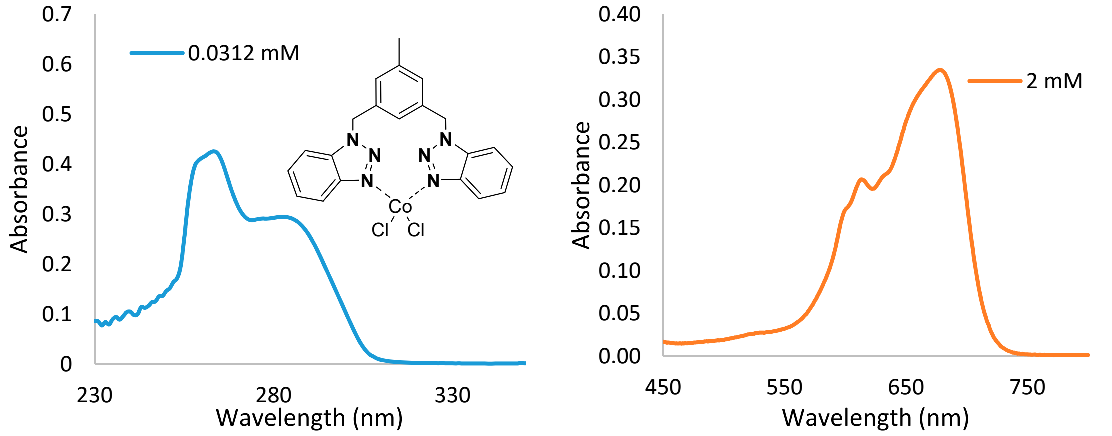

Figure S47. UV-Vis spectrum of dichloro[3,5-bis(benzotriazol-1-ylmethyl)toluene-NN]cobalt(II) in DMSO (11)

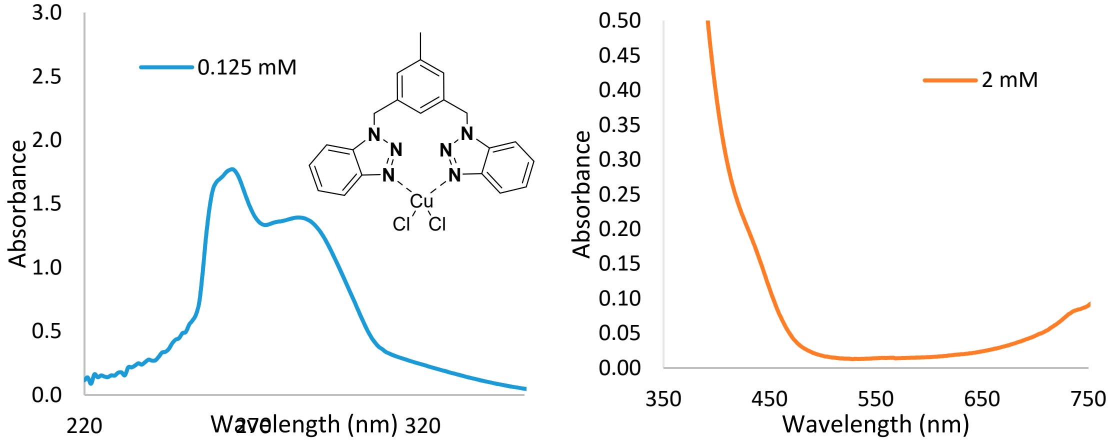

Figure S48. UV-Vis spectrum of dichloro[3,5-bis(benzotriazol-1-ylmethyl)toluene-NN]copper(II) in DMSO (12)

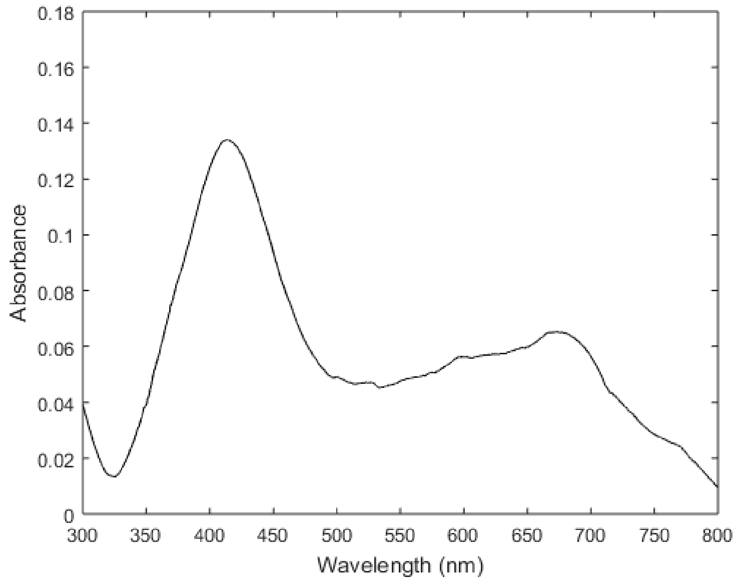

Figure S49. UV-Vis spectrum of AgNPs

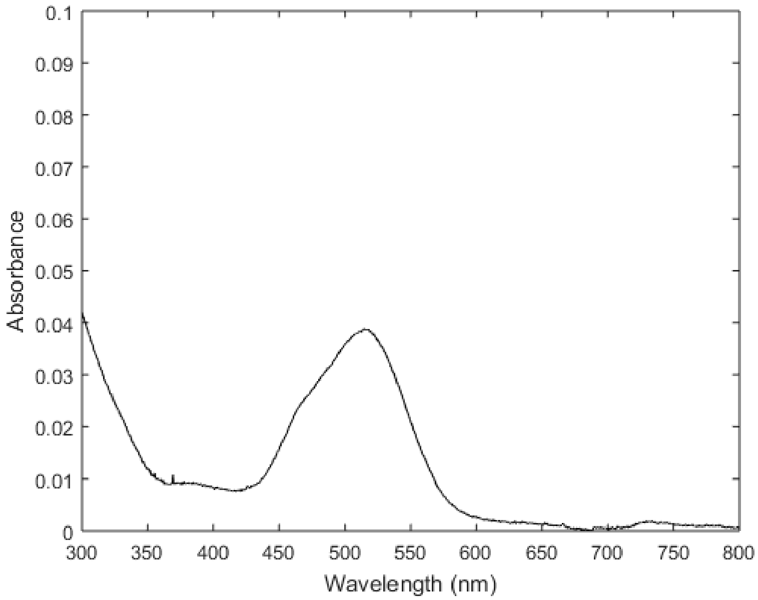

Figure S50. UV-Vis spectrum of **9**

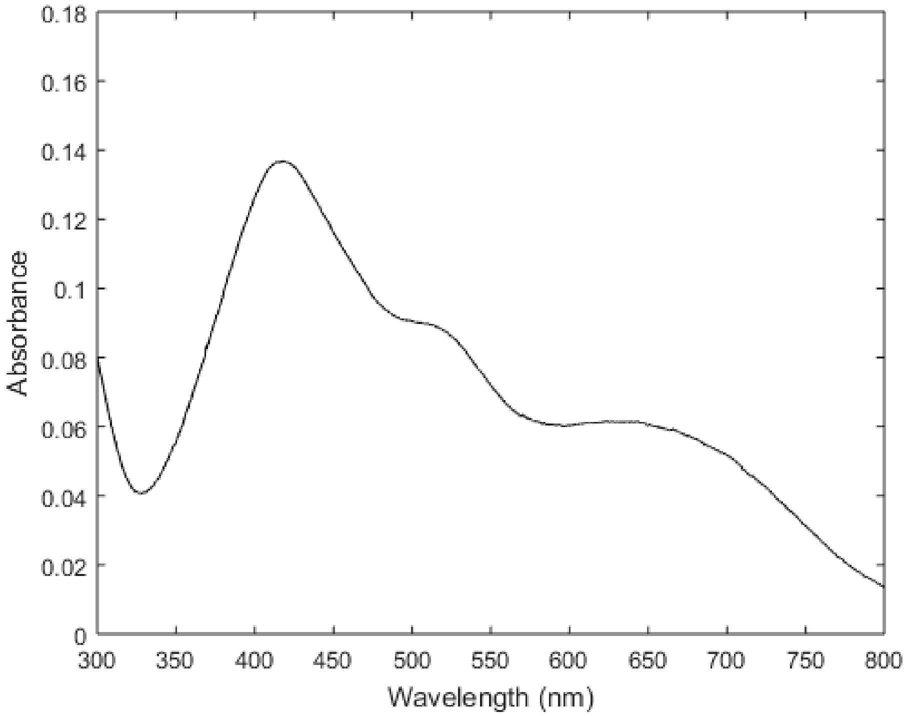

Figure S51. UV-Vis spectrum of **9** + AgNPS

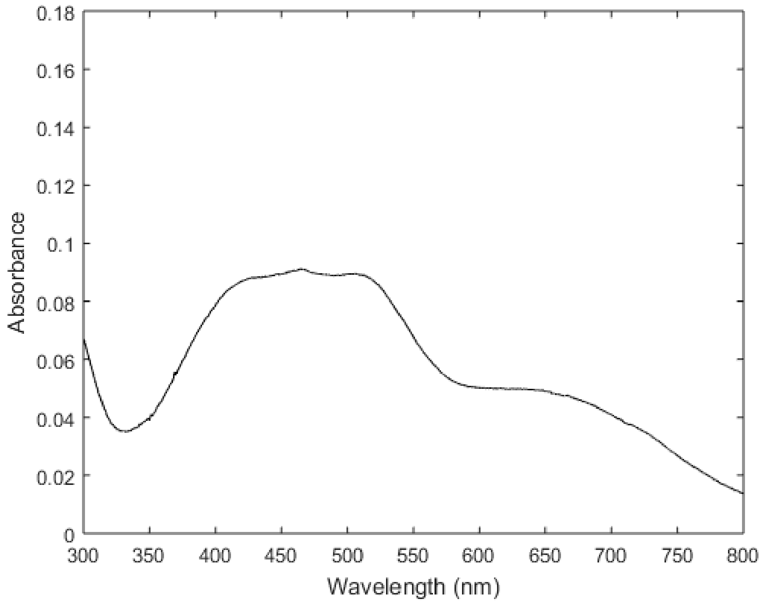

Figure S52. UV-Vis spectrum of **9** + AgNPS at 24h

Computational Calculations

Computational Details: Relativistic density functional theory calculations were carried out by using the ADF code, incorporating scalar (SR) corrections via the one-component ZORA Hamiltonian [6-8]. We employed all-electron triple-ξ Slater basis set, plus two polarization functions (STO-TZ2P) within the generalized gradient approximation (GGA) according to the Perdew-Burke-Ernzerhof (PBE) exchange-correlation functional because of its improved performance on long-range interactions and relatively low computational cost. Geometry optimizations were performed without any symmetry restrain, via the analytical energy gradient method implemented by Versluis and Ziegler. An energy convergence criterion of 10^{-4} Hartree, gradient convergence criteria of 10^{-3} Hartree/Å and radial convergence criteria of 10^{-2} Å were employed for the evaluation of the relaxed structures. The PBE functional at the scalar relativistic level and the Davidson method were employed in the TD-DFT calculations for calculate the optical properties.

The optimized geometries are given below (Figure S53), which agrees with the characterized structures, which leads to similar FTIR bands

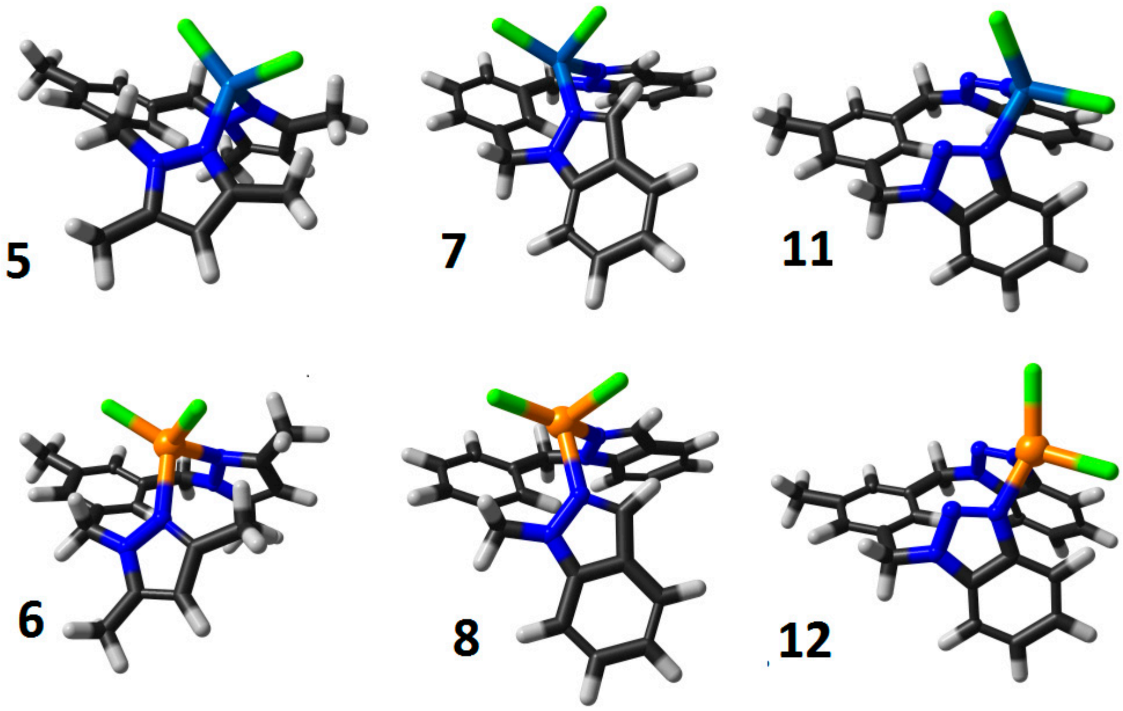

Figure S53. Optimized structure for the new compounds, **5, 6, 7, 8, 11** and **12**.

Table S1. Calculated and experimental main bands in the FTIR spectra for the complexes. Pyr = pyrazole; Ind = indazole; Tol = toluene.

**Compound**	**Wavenumber ν (cm⁻¹)**				
	(C-H)	(C-CH_3)	$(C\text{-}C)_{Pyr}$	(C-C-N)	(M-Cl)
**5exp**	3127	1468	1608	729	419
**5calc**	3010	1430	1597	682	394
**6exp**	3137	1469	1608	738	420
**6calc**	3108	1430	1593	760	481
	(C-H)	$(C\text{-}C)_{Ind}$	$(C\text{-}N)_{Ind}$	$(C\text{-}H)_{Ind}$	(M-Cl)
**7exp**	3094	1628	1519	1478	490
**7calc**	3105	1591	1501	1462	424

**8exp**	3098	1628	1519	1477	497
**8calc**	3115	1640	1498	1456	434
	(C-H)	$(C\text{-}C)_{Tol}$	(N-N)	(N=N)	(M-Cl)
**11exp**	2970	1610	1284	1229	419
**11calc**	3091	1596	1245	1205	425
**12exp**	2968	1610	1288	1233	419
**12calc**	3099	1590	1254	1215	427

References

1. Oki, A. R.; Sanchez, J.; Hamilton, S.; Emge, T. J. High-Spin Four and Six-Coordinate Cobalt(II) Complexes with 3,5-Dimethyl Pyrazole. J. Coord. Chem. 1995, 36, 63–69, doi:10.1080/00958979508022221.
2. Leovac, V. M.; Petković, R.; Kovács, A.; Pokol, G.; Szécsényi, K. M. Reactions of divalent transition metal halides with 3,5-dimethyl-1-(hydroxymethyl)-pyrazole. J. Therm. Anal. Calorim. 2007, 89, 267–275, doi:10.1007/s10973-006-7564-8.
3. Reedijk, J.; Verbiest, J. Coordination compounds derived from transition metal salts and bis(3,5-dimethylpyrazolyl)methane. Transit. Met. Chem. 1979, 4, 239–243, doi:10.1007/BF00619177.
4. Potapov, A. S.; Khlebnikov, A. I. Synthesis of mixed-ligand copper(II) complexes containing bis(pyrazol-1-yl)methane ligands. Polyhedron 2006, 25, 2683–2690, doi:10.1016/j.poly.2006.03.016.
5. Lobbia, G. G.; Bonati, F. Adducts Between Bis(1,2,4-Triazol-1-yl)Methane and First Row Transition Metal Halides or Nitrates. Synth. React. Inorg. Met.-Org. Chem. 1988, 18, 551–558, doi:10.1080/00945718808060811.
6. Amsterdam Density Functional (ADF) Code, Vrije Universiteit: Amsterdam, The Netherlands. Http://www.scm. Com.
7. van Lenthe, E.; Baerends, E.-J. J.; Snijders, J. G. Relativistic Total Energy Using Regular Approximations. J. Chem. Phys. 1994, 101 (11), 9783 doi: 10.1063/1.467943.
8. Wang, Y.; Burke, K.; Perdew, J. P. Generalized Gradient Approximation for the Exchange-Correlation Hole of a Many-Electron System. Phys. Rev. B 1996, 54, 16533–16539, doi: 10.1103/PhysRevB.54.16533
